# Probability via Expectation Measures

**DOI:** 10.3390/e27020102

**Published:** 2025-01-22

**Authors:** Peter Harremoës

**Affiliations:** GSK Department, Niels Brock, Copenhagen Business College, Nørre Voldgade 34, 1358 Copenhagen K, Denmark; harremoes@ieee.org

**Keywords:** category, double slit experiment, expectation measure, extended probabilistic power domain, expected value, Gaussian approximation, information divergence, monad, point process, Poisson distribution, Poisson point process, quantum information theory, thinning, valuation, 60A05, 60G55

## Abstract

Since the seminal work of Kolmogorov, probability theory has been based on measure theory, where the central components are so-called probability measures, defined as measures with total mass equal to 1. In Kolmogorov’s theory, a probability measure is used to model an experiment with a single outcome that will belong to exactly one out of several disjoint sets. In this paper, we present a different basic model where an experiment results in a multiset, i.e., for each of the disjoint sets we obtain the number of observations in the set. This new framework is consistent with Kolmogorov’s theory, but the theory focuses on expected values rather than probabilities. We present examples from testing goodness-of-fit, Bayesian statistics, and quantum theory, where the shifted focus gives new insight or better performance. We also provide several new theorems that address some problems related to the change in focus.

## 1. Introduction

Throughout the history of probability theory, some mathematicians have focused on probabilities, and others have focused on expectations. In his seminal paper from 1933, Kolmogorov focused on probabilities [1], but in the present paper, we will develop *expectation theory* as an alternative to probability theory, focusing on expectations. Expectation theory has been developed to handle technical problems in several applications in information theory, statistics (both frequentist and Bayesian), quantum information theory, and probability theory itself. We will present the basic definitions of our theory and provide interpretations of the core concepts. Standard probability theory, as developed by Kolmogorov, can be embedded in the present theory. Similarly, our theory can be embedded into standard probability theory. The focus is on discrete measures to keep this paper at a moderate length. Since there is no inconsistency between the two theories, there are many cases where expectation theory, as developed here for discrete measures, can also be used for more general measures. Some readers may be more interested in theory, and others may be more interested in applications. This paper is written hoping that the different sections can be read quite independently.

### 1.1. Organization of the Paper

In Section 2, we point out some significant ideas related to the work of Kolmogorov and we will review some later developments that are important for our topic. The notion of a probability monad will be presented in Section 2.3. This approach allows us to focus on which basic operations are needed to define a well-behaved class of models.

The theoretical framework we will develop may be viewed as a part of the theory of point processes. Usually, the topic of point processes is considered an advanced part of probability theory. Still, we will consider some aspects of the theory of point processes as quite fundamental for modeling randomness. In Section 2.4, we will give a short overview of the relevant concepts of the theory of point processes. For readers familiar with the theory of point processes, it puts our contribution in a well-known context. It will also provide a framework to ensure the rest of this paper provides a consistent theory. In the subsequent sections, our discussions will often focus on finite samples, but the conclusions will also hold for more general samples, which is easy to see with some general knowledge of point processes.

In Section 3, we develop the theory of finite empirical measures. Such finite empirical measures are formally equivalent to multisets. Some basic properties of empirical measures are established. It is pointed out that many problems in information theory can be formulated for empirical measures without reference to randomness.

In Section 4, we introduce finite expectation measures. In Section 4.3, we introduce the Poisson interpretation that allows us to translate between results for expectation measures and results for Poisson point processes with probability measures. The cost of this translation is that the outcome space of the process is infinite, even if the expectation measure is finite. The Poisson interpretation enables us to give probabilistic interpretations of conditioning and independence for general measures, as discussed in Section 4.4 and Section 4.5. In [2], it was demonstrated that the reverse information projection of a probability distribution into a convex set of probability distributions may not be a probability distribution. This has been an important motivation for studying information divergence for general measures, as done in Section 4.6.

In Section 5, we will provide examples of how the present theory gives alternative interpretations and improved results to some familiar problems in decision theory, Bayesian statistics, testing goodness-of-fit, and information theory.

We end the paper with a short conclusion, including a list of concepts in probability theory and the corresponding concepts in expectation theory.

### 1.2. Terminology

A measure with a total mass of 1 is usually called a probability measure or a normalized measure. We will deviate from this terminology and use the term *unital measure* for a measure with total mass 1. The term *normalized measure* will only be used when a unital measure is the result of dividing a finite measure by the total mass of the measure. We will reserve the word *probability measure* to situations where the weights of a unital measure are used to quantify uncertainty, and it is known that precisely one observation will be made and one can decide which event the observation belongs to in a system of mutually exclusive events that cover the whole outcome space. Similarly, we will talk about an *expectation measure* if our interpretation of its values are given in terms of expected values of some random variables. Other classes of measures are *coding measures* that are used in information theory and *mixing measures* that are unital measures used for barycentric decompositions of points in convex sets.

In standard probability theory, the probability measures live on a space often called a sample space, but we will use the alternative term, *an outcome space*. The word *sample* will be used informally about the result of a sampling process. The result of a point process will be called *an instance* of the process.

## 2. Probability Theory Since Kolmogorov

### 2.1. Kolmogorov’s Contribution

The modern foundation of probability theory is due to Kolmogorov. He contributed in many ways, and here we shall only focus on the aspects that are most relevant for the present paper. His 1933 paper [1] was in line with two ideas earlier mathematicians developed.

The first idea is symbolic logic, as developed by G. Boole. In this approach to logic, the propositions form a Boolean algebra. A *truth assignment function* is a binary function that assigns one of the values 0 (false) and 1 (true) to any proposition consistently. In particular, if *A* is a proposition, either *A* is assigned the value 1 or its negation ¬A is assigned the value 1. Kolmogorov’s work may be seen as an extension where statements are replaced by events, i.e., measurable sets, and any event *A* and its complement ∁A are assigned probabilities in $\left[0,1\right]$ in such a way that $P\left(A\right)+P\left(∁A\right)=1$. Thus, probability theory can be described as an extension of logic where the functions can take values in $\left[0,1\right]$ rather than values in {0,1}. Such extensions have been formalized as monads to be discussed in Section 2.3, but the theory of monads was only developed much later as part of category theory. See [3,4] for a general discussion of probability monads.

Lebesgue’s theory of measures inspired the second main idea in Kolmogorov’s 1933 paper. Measure theory was used to extend the previous definitions of integrals. The basic idea is that a measure is defined on a set of measurable sets. Such a measure should be countable additive like the notion of an area. Measure theory has been beneficial for the theory of integration, and in particular, it leads to compelling convergence theorems like Lebesgue’s theorem on dominated convergence. Kolmogorov used measure theory to allow for similar general convergence results in probability theory.

Basic probability theory is defined on measurable spaces, but several essential theorems do not hold for all measurable spaces. Therefore, it is often assumed that the outcome space is a standard Borel space. Such a space emerges if the measurable sets are the Borel sets of a topology defined by a complete separable metric space. This assumption will cover most applications. A standard Borel space has a one-to-one measurable mapping from the outcome space to the unit interval equipped with the Borel σ-algebra.

The primary objects in Kolmogorov’s probability theory are the outcome space and a probability measure on the outcome space. All probabilities are with respect to this outcome space and this probability measure. This assumption leads to a consistent theory, but it is just assumed that an outcome space and a probability measure exist. Theorems like the Daniell–Kolmogorov consistency theorem (also called Kolmogorov’s extension theorem ([5], page 246, Theorem 1)) make it quite explicit that the existence of an outcome space is a consistency assumption. For a random process $X={\left(\xi_{t}\right)}_{t\in T}$ where T⊆R, we define the *finite-dimensional distribution functions* by

(1) $$F_{t_{1},\ldots ,t_{n}}\left(x_{1},\ldots ,x_{n}\right)=P\left[\omega :\xi_{t_{1}}\leq x_{1},\ldots ,\xi_{t_{n}}\leq x_{n}\right]$$

defined for all sets with $t_{1}<t_{2}<\cdots <t_{n}$. For the random $X={\left(\xi_{t}\right)}_{t\in T}$ the finite-dimensional distributions function satisfies the *Chapman–Kolmogorov equations* stated below.

(2) $$\lim_{x_{k}\to \infty }F_{t_{1},\ldots ,t_{n}}\left(x_{1},\ldots ,x_{n}\right)=F_{t_{1},\ldots ,{\hat{t}}_{k},\ldots ,t_{n}}\left(x_{1},\ldots ,{\hat{x}}_{k},\ldots ,x_{n}\right)$$

where ∧ indicates an omitted coordinate

**Theorem** **1** (Kolmogorov’s Theorem on the existence of a process)**.** *Let $\left\{F_{t_{1},\ldots ,t_{n}}\left(x_{1},\ldots ,x_{n}\right)\right\}$, with $t_{i}\in T\subseteq R,t_{1}<t_{2}<\cdots <t_{n},n\geq 1$, be a given family of finite-dimensional distributions, satisfying the Chapman–Kolmogorov Equation (2). Then, there exists a probability space $\left(\Omega ,F,\phi \right)$ and a random process $X={\left(\xi_{t}\right)}_{t\in T}$ such that*

(3) $$P\left[\omega :\xi_{t_{1}}\leq x_{1},\ldots ,\xi_{t_{n}}\leq x_{n}\right]=F_{t_{1},\ldots ,t_{n}}\left(x_{1},\ldots ,x_{n}\right).$$

Later, category theory was developed, and commutative diagrams in category theory are exactly a language for expressing this type of consistency.

### 2.2. Probabilities or Expectations?

To a large extent, the present paper may be viewed as an extension of the point of view presented by Wittle [6]. By identifying an event with its indicator function, his exposition is formally equivalent to Kolmogorov’s probability theory.

If $X=\left(\Omega ,F\right)$ is a measurable space, we may define $F\left(\Omega ,F\right)$ as the set of bounded F-measurable functions Ω→R. For any unital measure μ on $F\left(\Omega ,F\right)$, we may define the functional $E_{\mu }:F\left(\Omega ,F\right)\to R$ by

(4) $$E_{\mu }\left(f\right)=\int_{\Omega }f\;d\mu .$$

The functional satisfies that

(5)Eμf≥0,whenf≥0;(6)Eμ1=1.

Any functional that satisfies these two conditions may be identified with a unital measure.

To describe weak convergence, we are interested in the outcome space as a topological space rather than as a measurable space. A second countable space is also a Lindelöf space, i.e., any open covering has a countable sub-covering. If the measure μ is locally finite, then the whole space has a covering by open sets of finite measures. In particular, the measure μ is σ-finite.

If the space is a locally compact Hausdorff space, then a locally finite measure is the same as a finite measure on compact sets. For such spaces, the integral

(7) $$\int_{\Omega }f\left(\omega \right)\;d\mu \omega$$

is well-defined for any function *f* that is continuous with compact support. Radon measures can be identified with positive functionals on $C_{c}\left(\Omega \right)$. With these conditions, the duality between Radon measures and continuous functions with compact support works perfectly without any pathological problems.

On a locally compact Hausdorff space, the finite measures can be identified with positive functionals on the continuous functions on the one-point compactification of the space.

### 2.3. Probability Theory and Category Theory

For the categorical treatment of probability theory, we first have to recall the definition of a transition kernel ([7], Chapter 1).

**Definition** **1.** *Let $\left(\Omega ,F\right)$ and $\left(S,S\right)$ be two measurable spaces. A* transition kernel* $\omega \to \mu_{\omega }$ from *Ω* to S is a function*

(8) $$\mu :\Omega \times S\to \left[0,\infty \right]$$

*such that:*

- *For any fixed B∈S the mapping*
  (9) $$\omega \to \mu_{\omega }\left(B\right)$$
  *is measurable.*
- *For every fixed ω∈Ω, the mapping*
  (10) $$B\to \mu_{\omega }\left(B\right)$$
  *is a measure on $\left(S,S\right)$.*

Let $M_{+}\left(\Omega ,F\right)$ denote the measures on $\left(\Omega ,F\right)$. If $P\in M_{+}\left(\Omega ,F\right)$, then a measure on $\left(S,S\right)$ is given by

(11) $$B\to \int_{\Omega }\mu_{\omega }\left(B\right)\;dP\omega .$$

Thus, a transition kernel may be identified with a mapping $M_{+}\left(\Omega ,F\right)\to M_{+}\left(S,S\right)$ that we will call a *transition operator*.

If the measure $\mu_{\omega }$ is a unital measure for any ω∈Ω, then the transition kernel is called a *Markov kernel*. The key observation for the categorical treatment of probability theory is that Markov kernels can be composed.

The first to put probability theory into the framework of category theory seems to be Lawvere [8]. He defined a category Pro that has measurable spaces as objects and Markov kernels as morphisms. The category Pro contains the singleton sets as initial objects. A probability measure on $\left(\Omega ,F\right)$ can then be identified with a morphism from an initial object to $\left(\Omega ,F\right)$.

Later, the theory of monads was introduced in category theory, and monads have had a significant impact on functional programming [9]. The first to describe the category Pro in terms of monads was Giry [10]. First, we consider the category of measurable spaces Meas. It has measurable spaces as objects and measurable maps between measurable spaces as morphisms.

To the measurable space $X=\left(\Omega ,F\right)$ we associate the set $M_{+}^{1}\left(X\right)$ of probability measures on *X*. The set $M_{+}^{1}\left(X\right)$ is equipped with the smallest σ-algebra such that the maps $f\to E_{\mu }\left(f\right)$ are all measurable where $E_{\mu }\left(f\right)=\int f\;dP$. If *g* is a measurable map from $X_{1}$ to $X_{2}$, then a measurable map $M_{+}^{1}\left(g\right)$ from $M_{+}^{1}\left(X_{1}\right)$ to $M_{+}^{1}\left(X_{2}\right)$ is defined by

(12) $$M_{+}^{1}\left(g\right)\left(E_{\mu }\right)\left(f\right)=E_{\mu }\left(f\circ g\right),$$

which can also be written as $M_{+}^{1}\left(g\right)\left(\phi \right)=E_{\mu }\circ F\left(g\right)$. If *f* is the indicator function of the measurable set *B* and the functional ϕ is given by the probability measure μ, then we obtain

(13) $$M_{+}^{1}\left(g\right)\left(\mu \right)\left(B\right)=\mu \left(g^{-1}\left(B\right)\right),$$

which is the usual definition of an *induced probability measure*. In this way, $M_{+}^{1}$ is a functor from the category Meas to itself, i.e., an *endofunctor*.

The morphisms in the category Pro are Markov operators $M_{+}^{1}\left(X_{1}\right)\to M_{+}^{1}\left(X_{2}\right)$, but Markov operators are given by Markov kernels. It is useful to describe in detail how one can switch between Markov operators and Markov kernels. For this purpose, we introduce a natural transformation δ that translates Markov operators into Markov kernels, and we introduce a natural transformation π that translates Markov kernels into Markov operators.

A measurable space $X=\left(\Omega ,F\right)$ can be embedded into $M_{+}^{1}\left(X\right)$ by mapping ω∈Ω into the Dirac measure $\delta_{\omega }$. In this way, $\delta_{\omega }\left(f\right)=f\left(\omega \right)$. Now, δ may be considered as a measurable map, i.e., a morphism in the category Meas. If id denotes the identity functor in Meas, then the following diagram commutes. 
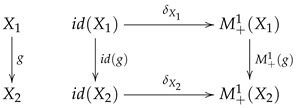
(14)
Thus, δ is a natural transformation from the identity functor id to the functor $M_{+}^{1}$. If $\Psi :M_{+}^{1}\left(X_{1}\right)\to M_{+}^{1}\left(X_{2}\right)$ is a Markov operator, then the corresponding Markov kernel $\psi :X_{1}\to M_{+}^{1}\left(X_{2}\right)$ is given by $\psi_{x}=\Psi \left(\delta_{x}\right)$.

We will use ${\left(M_{+}^{1}\right)}^{2}$ to denote the functor $M_{+}^{1}\circ M_{+}^{1}$. We have a measurable map π from ${\left(M_{+}^{1}\right)}^{2}\left(X\right)$ to $M_{+}^{1}\left(X\right)$ that maps $\mu \in {\left(M_{+}^{1}\right)}^{2}\left(X\right)$ to

(15) $$\pi \left(E_{\mu }\right)\left(f\right)=\int_{M_{+}^{1}\left(X\right)}E_{\nu }\left(f\right)\;d\mu \left(\nu \right).$$

Then, we have the following commutative diagram: 
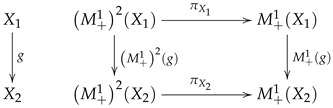
(16)
so that π is a natural transformation from ${\left(M_{+}^{1}\right)}^{2}$ to $M_{+}^{1}$. If $\psi :X_{1}\to M_{+}^{1}\left(X_{2}\right)$ is a Markov kernel, then the corresponding Markov operator Ψ is given by $\Psi =\pi \circ M_{+}^{1}\left(\psi \right)$. Thus, the following diagram commutes: 
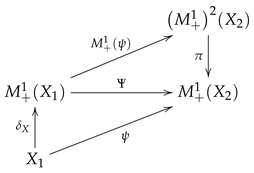
(17)

A Markov kernel from $X_{1}$ to $X_{2}$ may now be described as a measurable map $\psi_{1}$ from $X_{1}$ to $M_{+}^{1}\left(X_{2}\right)$. Composition of Markov kernels is given by

(18) $$\psi_{2}⊙\psi_{1}=\pi \circ M_{+}^{1}\left(\psi_{2}\right)\circ \psi_{1}$$

and we have the identities

(19)δ⊙ψ=ψ,(20)ψ⊙δ=ψ,(21)ψ1⊙ψ2⊙ψ3=ψ1⊙ψ2⊙ψ3.

The first two identities can be translated into the following commutative diagram: 
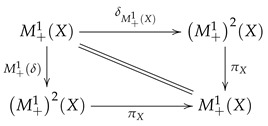
(22)
Whenever this diagram commutes, we say that δ *acts as an identity*. Associativity means that the following diagram commutes: 
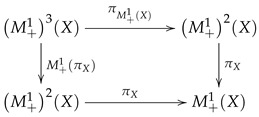
(23)
and we say that the functor $M_{+}^{1}$ is *associative*.

When an endofunctor $M_{+}^{1}$ together with two natural transformations δ and π satisfies associativity and δ acts as the an identity, we say that $\left(M_{+}^{1},\delta ,\pi \right)$ forms a *monad*. For any monad, morphisms from *X* to $M_{+}^{1}\left(X\right)$ can be composed by Equation (18) leading to the *Kleisli composition* of morphisms. For a category with a monad, the Kleisli category of the monad has the same objects as the original category, and as morphisms, it has the Kleisli morphisms. In this way, the category of Markov kernels can be identified with the Kleisli morphisms associated with the monad $\left(M_{+}^{1},\delta ,\pi \right)$. The Kleisli category is equivalent to the category introduced by Lawvere. The equivalence is established by a functor that maps the object *X* into $M_{+}^{1}\left(X\right)$ and maps Kleisli morphisms into their extensions.

### 2.4. Preliminaries on Point Processes

First, we will define a point process with points in *S*. Typically, *S* will be a *d*-dimensional Euclidean space, but *S* could, in principle, denote any complete separable metric space. Let S denote the Borel σ-algebra on *S*. Let $\left(\Omega ,F,P\right)$ denote a probability space. A transition kernel $\omega \to \mu_{\omega }$ from $\left(\Omega ,F\right)$ to $M_{+}\left(S,S\right)$ is called a *point process* if

- For all ω∈Ω the measure $\mu_{\omega }\left(\cdot \right):S\to R_{0,+}$ is locally finite.
- For all bounded sets B∈S the random variable $\omega \to \mu_{\omega }\left(B\right):\Omega \to R_{0,+}$ is a count variable.

For further details about point processes, see [11] or ([7], Chapter 3).

The interpretation is that, if the outcome is ω, then $\mu_{\omega }$ is a measure that counts how many points there are in various subsets of *S*, i.e., $\mu_{\omega }\left(B\right)$ is the number of points in the set B∈S. Each measure $\mu_{\omega }$ will be called *an instance* of the point process. In the literature on point processes, one is often interested in *simple point processes* where $\mu_{\omega }\left(B\right)=0$ when *B* is a singleton. However, point processes that are not simple are also crucial for the problems that will be discussed in this paper.

The definition of a point process follows the general structure of probability theory, where everything is based on a single underlying probability space. This will ensure consistency, but often this probability space has to be quite large if several point processes or many random variables are considered simultaneously.

The measure μ is called the *expectation measure* of the process $\omega \to \mu_{\omega }$ if for any B∈S we have

(24) $$\begin{array}{cc} \mu \left(B\right) & =\int_{\Omega }\mu_{\omega }\left(B\right)\;dP\omega . \end{array}$$

The expectation measure gives the mean value of the number of points in the set *B*. Different point processes may have the same expectation measure.

*A one-point process* is a process that outputs precisely one point with probability 1. For a one-point process the expectation measure of the process is simply a probability measure on *S*. Thus, probability measures can be identified with one-point processes. It is possible to define a monad for point processes [12]. The monad defined in [12] is also related to the observation that the Giry monad is distributive over the multiset monad as discussed in [13]. These results are closely related to the results we will present in the following sections.

A point process can be *thinned*, meaning each point in an instance $\mu_{\omega }$ is kept or deleted according to some random procedure. We can do α-thinning for $\alpha \in \left[0,1\right]$ by keeping a point in the sample with probability α and deleting it from the sample with probability 1−α. This is done independently for each point in the sample. Thus, $\omega \to \nu_{\omega }$ is an α-thinning of $\omega \to \mu_{\omega }$ if

(25) PνωB=νB=μBνBανB1−αμB−νB

for any measurable set *B*. If $\omega \to \nu_{\omega }$ is an α-thinning of $\omega \to \mu_{\omega }$, we write $\nu_{\omega }=T_{\alpha }\mu_{\omega }$.

### 2.5. Poisson Distributions and Poisson Point Processes

For $\lambda \in \left[0,\infty \right)$, the Poisson distribution $Po\left(\lambda \right)$ is the probability distribution on $N_{0}$ with point probabilities

(26) $$Po\left(j,\lambda \right)=\frac{\lambda^{j}}{j!}\exp\left(-\lambda \right).$$

The Poisson distribution may be viewed as a point process on a singleton set. The set of Poisson distributions is closed under addition and thinning.

(27)Poλ1∗Poλ2=Poλ1+λ2,(28)TαPoλ=Poα·λ.

For any s-finite measure μ on $\left(S,S\right)$ one can define a *Poisson point process* $\omega \to \mu_{\omega }$ with expectation measure μ ([14], Thm. 3.6). The following two properties characterize the Poisson point process.

- For all B∈S, the random variable $\omega \to \mu_{\omega }\left(B\right)$ is Poisson distributed with mean value $\mu \left(B\right)$.
- If $B_{1},B_{2}\in S$ are disjoint, then the random variables $\omega \to \mu_{\omega }\left(B_{1}\right)$ and $\omega \to \mu_{\omega }\left(B_{2}\right)$ are independent.

The Equations (27) and (28) also hold if the numbers λ are replaced by measures. The following result was essentially proved by Rényi [15] and Kallenberg [16].

**Theorem** **2.** *Let P be a unital measure and let $\omega \to \mu_{\omega }$ be independent point processes with expectation measure $\pi \left(P\right)=\mu$. Then, T1n∗i=1nμωi converges to $Po\left(\mu \right)$ in total variation.*

### 2.6. Valuations

Until now, we have presented the results in terms of measure theory. For a more general theory, it is helpful to switch to from measures to *valuations* [17]. Any measure μ satisfies the following properties:

**Strictness** $\mu \left(\emptyset \right)=0$.
**Monotonicity** For all subsets A⊆B, implies $\mu \left(A\right)\leq \mu \left(B\right)$.
**Modularity** For all subsets A,B,
  (29) $$\mu \left(A\right)+\mu \left(B\right)=\mu \left(A\cup B\right)+\mu \left(A\cap B\right).$$

For any lattice with a bottom element, a valuation is defined as a function that satisfies strictness, monotony, and modularity. The notion of a valuation makes sense in any lattice with a bottom element.

We are mainly interested in valuations that are continuous in the following sense. The possible values of a valuation are lower reals in $\vec{\left[0,\infty \right]}$, which are elements in the set $\left[0,\infty \right]$ with a topology of lower bounded open intervals. Thus, a function into $\vec{\left[0,\infty \right]}$ is continuous, if it is lower semicontinuous when $\left[0,\infty \right]$ is the usual topology.

**Continuity** $\mu \left(\sup_{\lambda }A_{\lambda }\right)=\sup_{\lambda }\mu \left(A_{\lambda }\right)$ for any directed net $A_{\lambda }$.

This notion of continuity captures both the inner regularity of a measure and it captures σ-additivity.

A Borel measure restricted to the open sets of a topological space is a valuation. On any complete separable metric space, any continuous valuation on the open sets is the restriction of a Borel measure. It will not make any difference whether we speak of measures or valuations for the applications that we will discuss in Section 5.

If *X* is a topological space, then V(X) denotes the set of continuous valuations on *X*. The set of valuations has a structure as a topological space, so that V is an endofunctor in the category Top of topological spaces. The functor V defines a monad that is called *the extended probabilistic power domain* [18]. It is defined in much the same way as the monads defined by Giry.

## 3. Observations

The outcome space plays a central role in Kolmogorov’s approach to probability. The basic experiment in his theory will result in a single point in the outcome space. The measurable subsets of the outcome space play the same role as the propositions play in a Boolean algebra in logic. The principle of excluded middle in logic states that any proposition is either true or false. Similarly, the basic experiment in Kolmogorov’s theory results in a single point, and these points exclude each other. In this paper, the result of a basic experiment will be a multiset rather than a single point.

### 3.1. Observations as Multiset Classifications

In computer science, we operate with different data types. *A set* is a collection of objects, where repetition and order are not relevant. *A list* is a collection of objects, where order and repetition are relevant. *Multisets* are unordered, but repetitions are relevant, so objects are counted with multiplicity. We shall discuss the relation between these data types in this subsection.

A review of the theory of multisets can be found in [19]. Monro [20] discusses two ways of defining a multiset, and the distinction between these definitions is important for the present work.

The following example is similar to what can be found in basic textbooks on statistics.

**Example** **1.** *Consider a list of observations of five animals like (cow,horse,bee,sheep,fly). The list may be represented by a table with a number as a unique key that indicates the order in which we have made the observations.*

$$\begin{array}{ll} \mathrm{Key} & \mathrm{Animal}\\ 1 & \mathrm{cow}\\ 2 & \mathrm{horse}\\ 3 & \mathrm{bee}\\ 4 & \mathrm{sheep}\\ 5 & \mathrm{fly} \end{array}$$

*In the present example, all the animals are different and if we are not interested in the order in which we have made the observations, we may represent the observations by the set Ω={bee,cow,fly,horse,sheep} where the animals have been displayed in alphabetic order, but in a set the order does not matter.*

*These animals can be classified as either mammals or insects, leading to the list of observations (mammal,mammal,insect,mammal,insect) or, equivalently, to the table.*

$$\begin{array}{ll} \mathrm{Key} & \mathrm{Animal}\\ 1 & \mathrm{mammal}\\ 2 & \mathrm{mammal}\\ 3 & \mathrm{insect}\\ 4 & \mathrm{mammal}\\ 5 & \mathrm{insect} \end{array}$$

*Since the list contains repetitions, we cannot represent it by the set A={insect,mammal}. Instead, we may represent it by the multiset $\left[insect,insect,mammal,mammal,mammal\right]$.*

According to the first definition of *a multiset* by Monro [20], a multiset is a set Ω with an equivalence relation ≃. If A denotes the set of equivalence classes, then we obtain a mapping g:Ω→A. Alternatively, any mapping g:Ω→A leads to equivalences classes on Ω. Dedekind was the first to identify a multiset with a function [19]. To each equivalence class, we assign the number of elements in the equivalence class. This is called the *multiplicity* of the equivalence class.

A category Mul with multisets as objects was defined by Monro [20]. An object in the category Mul is a set Ω with an equivalence relation ≃. If $\left(\Omega_{1},{≃}_{1}\right)$ and $\left(\Omega_{2},{≃}_{2}\right)$ are objects in the category Mul, then a morphism from $\left(\Omega_{1},{≃}_{1}\right)$ to $\left(\Omega_{2},{≃}_{2}\right)$ is a mapping $f:\Omega_{1}\to \Omega_{2}$ that respects the equivalence relations, i.e., if $x{≃}_{1}y$ in $\left(\Omega_{1},{≃}_{1}\right)$ then $f\left(x\right){≃}_{2}f\left(y\right)$ in $\left(\Omega_{2},{≃}_{2}\right)$. This category has been studied in more detail in [21].

An equivalence is often based on a partial pre-order. Let ⪯ denote a partial pre-ordering on Ω. Then, an equivalence relation ≃ is defined by a≃b if and only a⪯b and b⪯a. If A=ω/≃ then ⪯ induces a partial ordering on A, that we will also denote ⪯.

The downsets (hereditary sets) in $\left(\Omega ,⪯\right)$ form a distributive lattice with ∩ and ∪ as lattice operations. If the set A is finite, then the lattice is finite, and if $\left(A,⪯\right)$ satisfies the *descending chain condition* (DCC) then so does the lattice of downsets. Any finite distributive lattice can be represented by a finite poset ([22], Thm. 9), and a distributive lattice that satisfies DCC can be represented by a poset that satisfies DCC. There is a one-to-one correspondence between the elements of A and the irreducible elements of the lattice. This construction is an example of the construction of a *concept lattices* [23] based a relation, which in this case is the partial pre-ordering ⪯.

The partial pre-ordering ⪯ is an equivalence relation if and only if the lattice generated is a Boolean lattice. Therefore, we obtain a lattice where one cannot form complements except if ⪯ is an equivalence relation. The shift from Boolean lattices to more general lattices corresponds to shifting from logic with a law of excluded middle to more general classification systems.

The set of downsets in $\left(\Omega ,⪯\right)$ is closed under finite intersections and under arbitrary unions because any union is a finite union. Thus, the downsets in $\left(\Omega ,⪯\right)$ form a *topology*. The continuous functions for this topology are monotone functions for the ordering. Topological spaces and continuous functions form a category. If some of the equivalence classes in $\left(\Omega ,⪯\right)$ have more than one element, then the topology does not satisfy the separation condition $T_{0}$, but the topology on equivalence classes always satisfies $T_{0}$.

Multisets can also be described using σ-algebras as it is done in probability theory. A classification on Ω given by an equivalence relation ≃ or by a partial pre-ordering ⪯ leads to a topology on Ω. This topology generates a Borel σ-algebra on Ω. For any two outcomes $\omega_{1},\omega_{2}\in \Omega$ we have $\omega_{1}≃\omega_{2}$ if and only if $\omega_{1}\in B\Leftrightarrow \omega_{2}\in B$ for all Borel measurable sets *B*.

### 3.2. Observations as Empirical Measures

According to the second definition of *a multiset* discussed by Monro [20], a multiset is a mapping of a set A into $N_{0}$ that gives the multiplicity of each of the elements. This corresponds to the data types in statistics that are called *count data*. Here, we will represent such multisets by *finite empirical measures*.

**Example** **2.** *The list (mammal,mammal,insect,mammal,insect) can be written as a multiset $\left[insect,insect,mammal,mammal,mammal\right]$ or, equivalently, we may represent the multiset by the measure $2\cdot \delta_{insect}+3\cdot \delta_{mammal}$. Alternatively, the multiset can be represented by a table of frequencies.*

$$\begin{array}{ll} \mathrm{Classification} & \mathrm{Frequency}\\ \mathrm{insect} & 2\\ \mathrm{mammal} & 3 \end{array}$$

The mapping from lists to an empirical measure is called the *accumulation map*, and it is denoted Acc.

**Definition** **2.** *Let $\left(A,\tau \right)$ be a topological space. By a* finite empirical measure*, we understand a finite sum of Dirac measures on the Borel σ-algebra of $\left(A,\tau \right)$. The set of finite empirical measures on $\left(A,\tau \right)$ will be denoted $M\left(A,\tau \right)$ or $M\left(A\right)$ for short.*

We can perform the following operations with empirical measures:

- Addition.
- Restriction.
- Inducing.

The first operation we will look at is *addition*. Let $\ell_{1}$ and $\ell_{2}$ denote two lists of observations from the same alphabet A, and let $\ell_{1}*\ell_{2}$ denote the concatenation of the lists. Then, $Acc\left(\ell_{1}*\ell_{2}\right)=Acc\left(\ell_{1}\right)+Acc\left(\ell_{2}\right)$. Thus, the sum $\mu_{1}+\mu_{2}$ of two empirical measures has an interpretation via merging two datasets together. The corresponding operation for point processes is called *superposition*. Addition of empirical measures is a way of obtaining an empirical measure without using the accumulation map on a single list.

The next operation we will look at is *restriction*. If data are described by the empirical measure μ on A and *B* is a subset of A, then the restriction of μ to subsets of *B* is an empirical measure on *B* that we will denote by $\mu_{\cap B}$. In probability theory, all measures should be unital so we need the notion of conditional probability, but multisets cannot be normalized, and the concept simplifies to the notion of restriction.

Assume that g:A→B is a continuous (or measurable) mapping. Then, the induced measure $g\left(\mu \right)$ is defined by

(30) $$g\left(\mu \right)\left(B\right)=\mu \left(g^{-1}\left(B\right)\right),$$

where μ is an empirical measure on A and *B* is a measurable subset of B. The measure $M\left(g\right)\left(\mu \right)$ is called the *induced measure* and is often denoted $g\left(\mu \right)$ rather than $M\left(g\right)\left(\mu \right)$.

One can easily prove that inducing is additive in the measure and similar basic results regarding the interaction between addition, restriction, and creation of induced measures.

If Ω is a finite set and an equivalence on Ω is given by a function g:Ω→A, then the counting measure on A induces a finite empirical measure on A. Thus, the first definition of a multiset discussed by Monro [20] may be viewed as a special case of his second definition of a multiset. As our next examples will show, there are cases that the first definition of a multiset cannot handle but where the second definition works fine.

If the notion of observation is based on an equivalence, there is an implicit assumption that any two elements each have their own identity, but at the same time, they are equivalent according to a classification. In quantum physics, particles may be indistinguishable in a way that one would not see in classical physics.

**Example** **3.** *Young’s experiment, also called the double slit experiment, was first used by Young as a strong argument for the wave-like nature of light. Nowadays, it is often taken as an illustration of how quantum physics fundamentally differs from classical physics. The observations are often described as a paradox, but at least part of the paradox is related to a wrong presentation of the observations.*

**Figure 1:**
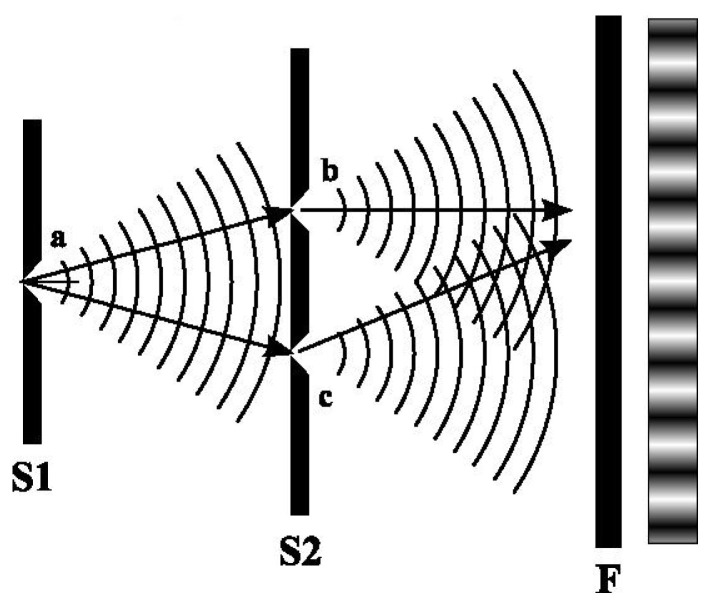
When coherent light is sent through first a single slit in screen S1 and then through the two slits b and c on screen S2, then an interference pattern emerges at the photographic film F.

*In its modern form, the experiment uses monochromatic light from a laser. The laser beam is first sent through a slit in a screen. The electromagnetic wave spreads like concentric circles after passing through the first slit, as illustrated in Figure 1. The wave then hits a second screen with two slits. After passing the two slits we obtain two waves that spread like concentric circles until they hit a photographic film that will display an interference pattern created by the two waves.*

*Now follows a "paradox" as it is described in many textbooks. The electromagnetic wave is quantized into photons, so if the intensity of the light is low we will only obtain separate points on the photographic film, but we obtain the same interference pattern as before. This may be explained as interference between photons passing through the left and the right slit, respectively. Now, we lower the intensity so much that the photons arrive at the photographic film one by one. After a long time of exposure, we obtain the same interference pattern as before. This apparently gives a paradox because it is hard to understand how a single photon should pass through both slits and have interference with itself.*

*A problem with the above description is that the number of photons emitted from a laser is described by a Poisson process. Even if the intensity is low, one cannot emit a single photon for sure. If the mean number of photons emitted is, say, 1, then there is still a probability that 0 or 2 (or more) photons are emitted and this will hold even if the intensity is very low. What we observe is a number of dots on the photographic film, which can be described as by a multiset if the photographic film is divided into regions. If we really want to send* single photons, *this could be achieved using* a single-photon source*. For a single-photon source there is no uncertainty in the number of photons emitted, but according to the time-energy uncertainty relation there will still be uncertainty in the energy. The energy E of a photon is given by*

(31) $$E=hf=h\frac{c}{\lambda }$$

*where h is Planck’s constant, f is the frequency, c is the speed of light, and λ is the wave length. Thus, uncertainty in the energy means uncertainty in frequency and wave length. Since the interference pattern observed on the photographic film depends on the wave length, one would not obtain the same interference pattern if a single-photon emitter was used instead of coherent light from a laser.*

*We see the idea that the observation in terms of a multiset comes from observation of a large number of* individual *photons is simply wrong and leads to an inconsistent description of the experiment.*

*To summarize: In Young’s experiment, we observe a multiset according to the second definition, i.e., the observation can be given in terms of a table containing the frequency of dots in various regions of the photographic film. If we try to describe the observations according to the first definition of a multiset, then we should label the individual photons something like photon 1, photon 2, etc., and then explain which of these individual photons resulted in a dot in certain regions of the photographic film. The first definition cannot be used because photons cannot be labeled.*

At first glance, the two definitions of a multiset appear to be equivalent, but the first definition is based on set theory, where the individual elements of a set can be labeled. Since we also want a theory useful for describing the results of quantum experiments like Young’s experiment, we prefer the second definition. In the previous example, indistinguishability was forced upon us by nature. In the next example, a kind of indistinguishability is desirable.

**Example** **4.** *If a man has 100 € and 50 $ in his wallet, we may represent it as $100\cdot \delta_{€}+50\cdot \delta_{\$}$. He may have 100 individual 1 € coins and 50 1 $ banknotes in the wallet. Alternatively, he may have two 50 € banknotes and one 50 $ banknote in his wallet. One cannot check if he has 100 € by just counting the coins. When we say that he has 100 € and 50 $, it means that he has something that is equivalent to having 100 individual 1 € coins and 50 individual 1 $ notes. Perhaps you borrow his two 50 € banknotes and later give him back two 50 € banknotes or one 100 € banknote. If he complains that he did not get back the individual 50 € banknotes that he gave you, he has not understood how money works. Money is a social construct! The social construct should work in such a way that having 100 € as physical individual coins or having the same amount as banknotes makes no difference for trading.*

The example with money could be extended because 1 € can be converted to 100 cents so that the measure may have decimal numbers as coefficients. Similarly, it even makes sense to allow negative coefficients in the description of savings and debt.

### 3.3. Categorical Properties of the Empirical Measures and Some Generalizations

Addition, restriction, and inducing can be described in the language of category theory. The existence of addition means that the category of multisets is *commutative*. The operation called restriction can be characterized as a *retraction* that is *additive*. The existence of induced measures means that M is a *functor* from topological spaces to measure spaces.

If $\left(A,\tau \right)$ is a topological space, then we equip $M\left(A,\tau \right)$ with the smallest topology such that

(32) μ→∫fdμ

are continuous for all continuous functions *f*. Here, the integral ∫fdμ simplifies to a sum $\sum_{i=1}^{n}f\left(a_{i}\right)$ when $\mu =\sum_{i=1}^{n}\delta_{a_{i}}$. If we define $M\left(g\right)\left(\mu \right)=g\left(\mu \right)$, then M becomes an endofunctor in the category of topological spaces and a monad can be defined in exactly the same way as for the Giry monad. Kleisli morphisms map points in a topological space into empirical measures on the topological space. Empirical measures will often be denoted $\mu_{\omega }$ in order to emphasize that an empirical measure typically is the result of some sampling process.

One can generalize finite empirical measures on a topological space to continuous integer valued valuations on a topological space. These integer valued valuations form a sub-monad of the monad of all continuous valuations.

As we have seen in Section 3.1, classifications may lead to other lattices than Boolean lattices, so it is relevant to discuss integer-valued valuations on more general distributive lattices. We shall work out the theory for finite lattices in all details below. If $\left(\Omega ,\tau \right)$ is a finite topological space, then we can define an integer-valued valuation *v* on the topology τ by

(33) $$v\left(B\right)=\#\left(B\right),$$

where $\#\left(B\right)$ means the number of elements in the open set B∈τ.

**Lemma** **1.** *Let v be a continuous valuation on a topological space $\left(\Omega ,\tau \right)$. Let c∈L be some element. Then, the restriction $v_{\cap c}$ given by $v_{\cap c}\left(a\right)=v\left(a\cap c\right)$ is an integer valued valuation. If v(c)<∞, then $v_{\setminus c}$ given by $v_{\setminus c}\left(a\right)=v\left(a\right)-v\left(a\cap c\right)$ is also an integer valued valuation and $v=v_{\cap c}+v_{\setminus c}$. If v is continuous, then $v_{\cap c}$ and $v_{\setminus c}$ are continuous.*

**Proof.**  The strictness of $v_{\cap c}$ and $v_{\setminus c}$ are obvious. The monotonicity of $v_{\cap c}$ is obvious. To see that $v_{\setminus c}$ is monotone, let a⊆b be some elements in the lattice. Then,

(34)va∪c≤vb∪c,(35)va+vc−va∩c≤vb+vc−vb∩c,(36)va−va∩c≤vb−vb∩c,(37)v∖ca≤v∖cb.

Modularity of $v_{\cap c}$ is a simple calculation. Modularity of $v_{\setminus c}$ follows because $v_{\setminus c}=v-v_{\cap c}$. Continuity is obvious.  □

Let *v* denote a valuation on a topological space $\left(\Omega ,\tau \right)$. Then, a∈L is called an *atom* of the valuation if b⊆a implies that v(b)=0 or v(b)=v(a). An *atomic valuation* is a valuation that is a sum of valuations for which *L* is an atom.

**Proposition** **1.** *Any integer valued valuation on a topological space $\left(\Omega ,\tau \right)$ is atomic.*

**Proof.**  The proof is by induction on n=v(Ω). First, the results hold for the trivial valuation with v(Ω)=0. Assume that the result holds for any valuation with v(Ω)≤n. Let *v* be a valuation with v(Ω)=n+1. If *L* is an atom, then *v* is atomic. If Ω is not an atom, then there exists c∈L such that 0<v(c)<v(Ω)=n. Then, $v_{\cap c}$ and $v_{\setminus c}$ are atomic valuations and $v=v_{\cap c}+v_{\setminus c}$, implying that *v* is atomic.  □

**Theorem** **3.** *Let $\left(A,\tau \right)$ denote a finite topological space. If the topology satisfies the separation condition $T_{0}$, then for any integer valued valuation v on the lattice of open sets there exists a uniquely determined empirical measure μ on A such that, for any open set B, we have*

(38) $$v\left(B\right)=\mu \left(B\right).$$

**Proof.**  We have to prove that if A is an atom for the valuation *v*, then *v* is given by a uniquely determined Dirac valuation. Let *A* denote a minimal atom. Then,

(39) $$\begin{array}{c} \begin{array}{cc} v(B) & =v(B\cup A)+v(B\cap A)-v(A)\\ \; & =v(A)+v(B\cap A)-v(A)\\ \; & =v(B\cup A)\\ \; & =\left\{\begin{array}{cc} v(A), & \;\mathrm{if}\;A\subseteq B;\\ 0, & \;\mathrm{else}. \end{array}\right. \end{array} \end{array}$$

For a∈A, let $a_{↓}$ denote the smallest open set that contains *a*. Then, there must exist a∈A such that $v\left(a_{↓}\right)=v\left(V\right)$, since, otherwise, one would have $V\left(A\right)=0.$. Hence, we have $A\subseteq a_{↓}$, which implies that $A=a_{↓}$. Hence,

(40) $$\begin{array}{cc} v(B) & =\left\{\begin{array}{cc} v(A), & \;\mathrm{if}\;a\in B;\\ 0, & \;\mathrm{else}. \end{array}\right.\\ \; & =\delta_{a}\left(B\right). \end{array}$$

Assume that $\delta_{a_{1}}=\delta_{a_{1}}$. Then, $\delta_{a_{1}}\left(B\right)=\delta_{a_{1}}\left(B\right)$ for all open sets *B*. Hence, $a_{1}\in B$ if and only if $a_{2}\in B$. Since the topology satisfies $T_{0}$, we must have $a_{1}=a_{2}$.  □

As a consequence of the theorem, any integer-valued valuation *v* on a finite distributive lattice can be represented by a finite set Ω with a topology such that the multiset obtained by mapping Ω to equivalence classes A satisfies (33).

For applications in statistics, the main example of a lattice is the topology of a complete separable metric space.

**Theorem** **4.** *Let μ denote an integer valued valuation on the topology of a complete separable metric space. Then, μ is a simple valuation, i.e., there exists integers $s_{1},s_{2},\ldots ,s_{n}\in N_{0}$ and letters $a_{1},a_{2},\ldots ,a_{n}\in A$ such that*

(41) $$\mu =\sum_{i=1}^{n}s_{i}\cdot \delta_{a_{i}}.$$

**Proof.**  Based on a result of Topsøe ([24], Thm. 3), Manilla has proved that any valuation on the topology of a metric space can be extended to a measure μ on the σ-algebra of Borel sets of the metric space ([25], Cor. 4.5). When the metric space is separable and complete, we may, without loss of generality, assume that the metric space is $B=\left[0,1\right]$ and let B denote the identity on $\left[0,1\right]$. Then, $F\left(x\right)=\mu \left(X\leq x\right)$ is increasing and integer-valued. Therefore, *F* is a staircase function with a finite number of steps. The measure *P* will have a point mass at each step and no mass in between. Hence, μ is simple.  □

### 3.4. Lossless Compression of Data

Bayesian statistics focus on single outcomes of experiments and the frequentist interpretation focus on infinite i.i.d. sequences. Information theory takes a position in between. In information theory, the focus is on extendable sequences rather than on finite or infinite sequences [26]. In lossless source coding, we consider a sequence of observations in the source alphabet A, i.e., an observation is an element in $A^{n}$ where *n* is some natural number. We want to encode the letters in the source alphabet by sequences of letters in an output alphabet B of length β. In lossless coding, the encoding should be uniquely decodable. Furthermore, the encoding should be so that as concatenation of source letters is encoded as the corresponding concatenation of output letters. We require that not only $A^{n}$ is uniquely decodable, but any (finite) string in $A^{*}$ should be encoded into a string in $B^{*}$ in a unique way. If the code $\kappa :A\to B^{*}$ is uniquely decodable, then it satisfies Kraft’s inequality

(42) $$\sum_{a\in A}\beta^{-\ell \left(\kappa \left(a\right)\right)}\leq 1$$

where $\ell \left(\kappa \left(a\right)\right)$ denotes the length of the code word $\kappa \left(a\right)$ ([27], Thm. 5.2.1). Instead of encoding single letters in A into $B^{*}$, we may do it as block coding where a block in $A^{n}$ is mapped as a string $B^{*}$ via a mapping κ. The following theorem is a kind of reverse of Kraft’s inequality for block coding.

**Theorem** **5** ([26]). *Let ℓ:A→R denote a function. Then, the function ℓ satisfies Kraft’s inequality (42) if and only if for all ϵ>0 there exists an integer n and a uniquely decodable code $\kappa :A^{n}\to B^{*}$ such that*

(43) $$\left|{\bar{\ell }}_{\kappa }\left(a_{1}^{n}\right)-\frac{1}{n}\sum_{i=1}^{n}\ell \left(a_{i}\right)\right|\leq \epsilon$$

*where ${\bar{\ell }}_{\kappa }\left(a_{1}^{n}\right)$ denotes the length $\ell_{\kappa }\left(a_{1}^{n}\right)$ divided by n.*

Using this theorem, we can identify uniquely decodable codes with code length functions that satisfy Kraft’s inequality. There is a correspondence between codes and sub-unital measures given by

(44) $$\mu \left(a\right)=\beta^{-\ell \left(\kappa \left(a\right)\right)}.$$

Now, the goal in lossless source coding is to code with a code-length that is as short as possible. If a code word has empirical measure μ and the function *ℓ* is used, then the total code length is

(45) $$\sum_{a}\ell \left(a\right)\mu \left(a\right).$$

Our goal is to minimize the total code length and this is achieved by the code length function

(46) $$\ell \left(a\right)=-\log_{\beta }\left(\frac{\mu (a)}{n}\right).$$

If a code with this code length function is used, then the total code length will be $n\cdot H\left(\frac{\mu }{n}\right)$ where *H* denotes the Shannon entropy of a probability measure. We can define the entropy of any finite discrete measure by

(47) $$H\left(\mu \right)=\mu \left(A\right)H\left(\frac{\mu }{\mu \left(A\right)}\right).$$

With this definition, one can easily prove that, if g:A→B is a measurable mapping and μ is a measure on A, then the following *chain rule* holds:

(48) $$H\left(\mu \right)=H\left(g\left(\mu \right)\right)+\sum_{b\in B}H\left(\mu_{|g^{-1}\left(b\right)}\right).$$

The chain rule reflects that coding the result in A can be performed by first coding the result in B and then coding the result in A restricted to subset of letters in A that maps into the observed letter in B.

One could proceed on exploring the correspondence between measures and codes as is done in the minimum description length (MDL) approach to statistics, and much of the content of Section 4 could be treated as using MDL. For instance, the number of source letters of length *n* is $\alpha^{n}$ and it grows exponentially, but the number of different multisets of size *n* is upper bounded by ${\left(n+1\right)}^{\alpha }$ and it grows like a polynomial in *n*. This fact has important consequences related to the maximum entropy principle and in the information theory literature [28] it is called the *method of types* where type is another word for multiset.

In order to emphasize the distinction between probability measures and expectation measures, we will not elaborate on this approach in the present exposition. Here, we will just emphasize that Kraft’s inequality and Equation (46) are some of the few examples where a theorem states that unital measure play a special role beyond the fact that all finite measures can be normalized.

### 3.5. Lossy Compression of Data

If an information source is compressed to a rate below the entropy, the source letter cannot be reconstructed from the output letters. In this case, one will experience a loss in description of the source and methods for minimizing the loss are handled by rate distortion theory. In rate distortion theory, we introduce a distortion function $d:A\times \hat{A}\to R$ that quantifies how much is lost if a∈A was sent, and it was reconstructed as $\hat{A}$. If $\hat{A}\subseteq A$, then *d* may be a metric or a function of a metric. Many aspects of statistical analysis can be handled by rate distortion theory (see [29] and references therein). This involves cluster analysis, outlier detection, testing goodness-of-fit, estimation, and regression. Important aspects of statistics can be treated using rate distortion theory, but in order to keep the focus on the distinction between probability measures and expectation measures, we will not go into further details regarding rate distortion theory.

## 4. Expectations

Multisets, empirical measures, and integer valued valuations are excellent for descriptive statistics, but these concepts neither describe randomness, sampling, nor expectations. In this section, we will discuss more general categories where these concepts are incorporated.

### 4.1. Simple Expectation Measures

Let Ω denote a large outcome space and let $\mu_{\omega }$ denote the empirical measure on the set A if the outcome is ω. The empirical measure $\mu_{\omega }$ can be described as a list of frequencies or as a multiset. Assume that the size of the multiset is $N=\mu_{\omega }\left(A\right)$. The measure $\mu_{\omega }$ may describe a sample from a population, but it may also describe the whole population, in which case the subscript ω is not needed. In modern statistics, various resampling techniques play an important role, and for this reason we will keep the subscript in order both to describe sampling and resampling. Now, we take a sample of size *n* from the population.

The simplest situation is when n=1. If B⊆A then $\frac{1}{N}\cdot \mu_{\omega }\left(B\right)$ is the probability that a randomly selected point from the multiset described by $\mu_{\omega }$ belongs to *B*. The unital measure $\frac{1}{\mu_{\omega }\left(A\right)}\cdot \mu_{\omega }$ is the *empirical distribution*. The empirical measure $\mu_{\omega }$ gives a table of frequencies and the empirical distribution gives a table of relative frequencies.

Next, consider the situation when we take a sample of size n>1 from the population. There are different ways of taking a sample of size *n*. One may sample with replacement or without replacement. These two basic sampling schemes are described by the multinomial distribution and by the multivariate hyper-geometric distribution, respectively. For both sampling methods, the mean number of observations in a set *B* is given by $\frac{n}{N}\cdot \mu_{\omega }\left(B\right)$. Thus, the expected values are described by the measure α·μ where α=nN. Here, we have scaled the measure $\mu_{\omega }$ by a factor $\alpha \in \left[0,1\right]$, and this leads to a measure that is not given by a multiset.

Consider a sample described by an empirical measure $\mu_{\omega }$ with sample size $N=\mu_{\omega }\left(A\right)$. For cross validation, we may randomly partition the sample into a number of subsamples. One may then check if a conclusion based on a statistical analysis of one of the subsamples is the same as if another subsample had been taken. If there are *k* random subsamples, then the expected number of observations in *B* is $\frac{1}{k}\cdot \mu_{\omega }\left(B\right)$ and the random subsample may be described by the measure $\alpha \cdot \mu_{\omega }$ where α=1k.

In bootstrap re-sampling, one selects *n* objects from a sample of size *n*, but this is done with replacement. If the sample is described by the measure $\mu_{\omega }$, then the mean number of observations in *B* is $\mu_{\omega }\left(B\right)$. Thus, bootstrap re-sampling corresponds to the measure $\alpha \cdot \mu_{\omega }$ where α=1. We see that, although multiplying by 1 does not change the measure, it may reflect a non-trivial re-sampling procedure.

In general, we may perform α-thinning of a multiset. This is done by keeping each point with probability α and deleting it with probability 1−α. The preservation/deletion of observations is performed independently for each observation. For values of α in $\left[0,1\right]\cap Q$, we can implement α-thinning using a random number generator that gives a uniform distribution on a finite set. Such random numbers can be created by rolling a die, draw a card from a deck, or a similar physical procedure. The set $\left[0,1\right]\cap Q$ is not complete, which is inconvenient for formulating various theorems. Therefore, we will also allow irrational values of α so that α can assume any value in $\left[0,1\right]$.

We discussed the addition of measures in the previous section. In particular, we may add *n* copies of the measure $\mu_{\omega }$ together to obtain the measure $n\cdot \mu_{\omega }$. Then, we may sample from $n\cdot \mu_{\omega }$ by thinning by some factor $\alpha \in \left[0,1\right]$ so that we obtain the measure $\alpha \cdot \left(n\cdot \mu_{\omega }\right)=\left(\alpha \cdot n\right)\cdot \mu_{\omega }$. In this way, we may multiply a measure by any positive number. In general, there will be many different ways of implementing a multiplication by the positive number *t*:

- There are many ways of writing *t* as a product α·n where $\alpha \in \left[0,1\right]$ and *n* is an integer.
- There are many different sampling schemes that will lead to a multiplication be α.
- There are many ways of generating the randomness that is needed to perform the sampling.

Although there are many ways of implementing a multiplication of the measure $\mu_{\omega }$ by the number t≥0, it is often sufficient to know the resulting measure $t\cdot \mu_{\omega }$ rather than details about how the multiplication is implemented. In Section 4.3, we will introduce a kind of default way of implementing the multiplication.

By allowing multiplication by positive numbers, we can obtain any finite measure concentrated on a finite set, i.e., measures of the form

(49) $$\mu =\sum s_{a}\cdot \delta_{a}.$$

The set $M_{+}^{fin}\left(A\right)$ is defined as the finitely supported finite measures on A. The finitely supported finite measures are related to the empirical measures in exactly the same way as probability measures are related to truth assignment functions in logic.

### 4.2. Categorical Properties of the Expectation Measures and Some Generalizations

We want to study a monad that allows us to work with both empirical measures and unital measures as done in probability theory. The set $M_{+}^{fin}\left(A\right)$ is defined as the finitely supported finite measures on A. If $P\in M_{+}^{fin}\left(M\left(A\right)\right)$ is a probability measure and the outcome space is $\Omega =M\left(A\right)$, then $\omega \to \mu_{\omega }$ is formally a point process with points in A. The expectation measure $\pi \left(P\right)$ of the point process $\omega \to \mu_{\omega }$ is given by $M_{+}^{fin}\left(M\left(A\right)\right)$ to $M_{+}^{fin}\left(A\right)$ by

(50) $$\pi \left(P\right)\left(f\right)=\int_{M\left(A\right)}\sum_{A}f\left(a\right)\mu_{\omega }\left(a\right)\;dP\omega .$$

By linearity, the transformation π defined by Equation (50) extends to a natural transformation from $M_{+}^{fin}\left(M_{+}^{fin}\left(A\right)\right)$ to $M_{+}^{fin}\left(A\right)$.

As before, we let δ denote the natural transformation that maps a∈A into $\delta_{a}$, i.e., the Dirac measure concentrated in *a*. It is straight-forward to check that $\left(M_{+}^{fin},\delta ,\pi \right)$ forms a monad. The Kleisli morphisms generated by this monad will generate a category that we will call *the finite expectation category*.

Finite lattices can be represented by finite topological spaces, and for these spaces the theory is simple.

**Theorem** **6.** *Let $\left(A,\tau \right)$ denote a finite topological space. If the topology satisfies the separation condition $T_{0}$, then for any finite valuation v on τ there exists a finite expectation measure μ on the Borel σ-algebra A such that for any open set B we have*

(51) $$v\left(B\right)=\mu \left(B\right).$$

**Proof.**  The proof is an almost a word-by-word repetition of the proof of Theorem 3.  □

All finite expectation measures are continuous valuations on a topological space. The monad of continuous valuations on topological spaces is important because it includes all probability measures on complete separable metric spaces, that is the most used model in probability theory.

### 4.3. The Poisson Interpretation

Let μ denote a discrete measure such that

(52) $$\mu =\sum_{a\in A}s_{a}\cdot \delta_{a}$$

where $A=\{a|\mu \left(a\right)>0\}$. Then, the Poisson point process $Po\left(\mu \right)$ given by the product

(53) $$\underset{a\in A}{⨂}Po\left(s_{a}\right)$$

is a point process with expectation measure μ. Thus, any discrete measure has an interpretation as the expectation measure of a discrete Poisson point process (see Section 2.5). This interpretation will be called *the Poisson interpretation*, and it can be used to translate properties and results for (non-unital) expectation measure into properties and results for probability measures.

A. Rényi was the first to point out that a Poisson process has extreme properties related to information theory [30] and entropy for point processes were later studied by McFadden [31]. Their results were formulated for simple point processes. Here, we will look at some results for processes supported on a finite number of points. If we thin a one-point process, we will obtain a process given by the expectation measure $P=\sum_{i}p_{i}\cdot \delta_{i}$ where $\sum_{i}p_{i}\leq 1$ and $1-\sum_{i}p_{i}$ is *the void probability*, i.e., the probability of obtaining no point. Here, we shall just present two results that are generalizations of similar results in [32,33]. We say that a point process is a *multinomial sum* if it is a sum of independent thinned one point processes. Let $Be\left(\mu \right)$ denote the set of sums of independent thinned one-point processes with expectation measure μ and let $Be^{*}\left(\mu \right)$ denote the total variation closure of $Be\left(\mu \right)$. As a consequence of Theorem 2, the distribution $Po\left(\mu \right)$ lies in $Be^{*}\left(\mu \right)$. The following results can be proved in the same way as a corresponding result in [32] was proved.

**Theorem** **7.** *The maximum entropy process in $Be^{*}\left(\mu \right)$ is the Poisson point process $Po\left(\mu \right).$*

The following result states that a homogeneous process has greater entropy than an inhomogeneous process with the same mean number of points. This is a point process version of the result that the uniform distribution is the distribution with maximal entropy on a finite set.

**Theorem** **8.** *Let A be a set with m elements. Then, the Poisson point process $Po\left(\mu \right)$ that has maximum entropy under the condition that $\mu \left(A\right)=\lambda$ is the process where μa=λm for all a∈A.*

**Proof.**  We note that

(54) $$\begin{array}{c} \begin{array}{cc} H\left(Po\left(\mu \right)\right) & =H\left(\underset{a}{⨂}Po\left(\mu \left(a\right)\right)\right)\\ \; & =\sum_{a}H\left(Po\left(\mu \left(a\right)\right)\right) \end{array} \end{array}$$

so it is sufficient to prove that the sum is Shur concave under the condition that $\sum_{a}\mu \left(a\right)=\lambda .$

Let $\mu_{1},\mu_{2}>0$ and let $X_{i}∼Po\left(\mu_{i}\right)$, then

(55) $$\begin{array}{c} H\left(X_{1}\right)+H\left(X_{2}\right)=H\left(\left(X_{1},X_{2}\right)\right)=H\left(X_{1}+X_{2}\right)+H\left(\left(X_{1},X_{2}\right)|X_{1}+X_{2}\right)\\ \;\;\;\;=H\left(Po\left(\mu_{1}+\mu_{2}\right)\right)+\sum_{n=0}^{\infty }H\left(bin\left(n,\frac{\mu_{1}}{\mu_{1}+\mu_{2}}\right)\right)\cdot \frac{{\left(\mu_{1}+\mu_{2}\right)}^{n}}{n!}\exp\left(-\left(\mu_{1}+\mu_{2}\right)\right), \end{array}$$

where $bin\left(n,p\right)$ means the binomial distribution with number parameter *n* and success probability *p*. For a fixed value of $\mu_{1}+\mu_{2}$, we have to maximize $H\left(bin\left(n,\frac{\mu_{1}}{\mu_{1}+\mu_{2}}\right)\right)$. The maximum is achieved when $\frac{\mu_{1}}{\mu_{1}+\mu_{2}}=\frac{1}{2}$, i.e., $\mu_{1}=\mu_{2}$. To see that $H\left(bin\left(n,p\right)\right)$ is maximal for p=12, it is sufficient to note that $p\to H\left(bin\left(n,p\right)\right)$ is a concave function which follows from [34].  □

It is also possible to construct Poisson point processes on finite topological spaces.

**Theorem** **9.** *Let v denote a valuation on the topology of a finite set A. Assume that the topology satisfies the separation condition $T_{0}$. Then, there exists an outcome space *Ω* with a probability measure P and a transition kernel $\omega \to \mu_{\omega }$ from *Ω* to A such that*

- *$\mu_{\omega }\left(B\right)$ is Poisson distributed for any open set B.*
- *For any open sets A,B,C the random variable $\mu_{\omega }\left(A\right)$ is independent of the random variable $\mu_{\omega }\left(B\right)$ given the random variable $\mu_{\omega }\left(C\right)$ if and only if A∩B⊂C.*

*Furthermore, for any open set B, we have*

(56) $$v\left(B\right)=\int_{\Omega }\mu_{\omega }\left(B\right)\;dP\omega .$$

**Proof.**  First, we determine the measure μ on A such that $v\left(B\right)=\mu \left(B\right)$ for any open set *B*. Then, we construct independent Poisson distributed random variables $X_{a}$ such that $X_{a}∼Po\left(\mu \left(a\right)\right)$. Then, for any open set *B*, we define a random variable

(57) $$Y_{B}=\sum_{a\in B}X_{a}.$$

With these definitions

(58) $$\begin{array}{c} \begin{array}{cc} E\left(Y_{B}\right) & =E\left(\sum_{a\in B}X_{a}\right)\\ \; & =\sum_{a\in B}E\left(X_{a}\right)\\ \; & =\sum_{a\in B}\mu \left(a\right)\\ \; & =\mu \left(B\right)\\ \; & =v\left(B\right). \end{array} \end{array}$$

The conditional independence follows from the construction.  □

It is worth noting that, for any lattice, the relation A∧B≤C defines a separoid relation (abstract notion of independence) if and only the lattice is distributive. For a distributive lattice, the relation A∧B≤C can also be written as $\left(A\vee C\right)\wedge \left(B\vee C\right)=C$, and this relation is separoid if and only the lattice is modular (see [35,36], Cor. 2).

### 4.4. Normalization, Conditioning, and Other Operations on Expectation Measures

Empirical measures can be added, one can take restrictions, and one can find induced measures. Using the same formulas, these operations can be performed on expectation measures, but we are not only interested in the formulas but also in probabilistic interpretations.

First, we note that any σ-finite measure μ is the expectation measure of some point process. Assume that $\mu =\sum_{i=1}^{\infty }\mu_{i}$ for some finite measures $\mu_{i}$. Then,

(59) $$\mu =\sum_{i=1}^{\infty }\frac{1}{2^{i}}\cdot \nu_{i}$$

where $\nu_{i}=2^{i}\cdot \mu_{i}$ are finite measures. Thus, μ is a probabilistic mixture of finite measures. Therefore, we just have to prove that any finite measure ν is the expectation measure of a point process. The norm of a (positive) measure ν is defined by $∥\nu ∥=\nu \left(A\right)$, and the *normalized measure*$\nu /∥\nu ∥$ has an interpretation as a probability measure, which is the same as a one-point process. We may add *n* copies of this process to obtain a process with expectation measure $n\cdot \nu /∥\nu ∥$. If $n\geq ∥\nu ∥$, then this process can be thinned to obtain a process with expectation measure ν. The following proposition gives probabilistic interpretations of addition, restriction, and inducing for expectation measures via the same operations applied to empirical measures. These equations are proved by simple calculations.

**Proposition** **2.** *Let $\left(\Omega ,F,P\right)$ be a probability space. Let $\omega \to \mu_{\omega }$ and $\omega \to \nu_{\omega }$ denote point processes with expectation measures μ and ν and with points in A. Let A be a subset of A and let f:A→B be some mapping. Then,*

(60)μ+ν=∫μω+νωdPω,(61)μ∩A=∫μω∩AdPω,(62)fμ=∫fμωdPω.

*respectively.*

Unital measures are normally called probability measures, and our aim is to give a probabilistic interpretation of the normalized measure $\frac{1}{∥\mu ∥}\cdot \mu$ by specifying an event that has probability equal to $\frac{\mu \left(B\right)}{∥\mu ∥}$.

**Theorem** **10.** *Let B be a subset of A. Let P denote a probability measure on *Ω* and assume that $\omega \to \mu_{\omega }$ is a Poisson point process with expectation measure μ. Then,*

(63) $$\frac{\mu \left(B\right)}{∥\mu ∥}=\int_{\Omega }\frac{\mu_{\omega }\left(B\right)}{∥\mu_{\omega }∥}\;dP\left(\omega |∥\mu_{\omega }∥>0\right).$$

**Proof.**  We take the mean of the empirical distribution with respect to *P*.

(64) $$\begin{array}{c} \begin{array}{cc} \int_{\Omega }\frac{\mu_{\omega }\left(B\right)}{∥\mu_{\omega }∥} & dP\left(\omega |∥\mu_{\omega }∥>0\right)\\ \; & =\sum_{n=1}^{\infty }\left(\int_{\Omega }\frac{\mu_{\omega }\left(B\right)}{∥\mu_{\omega }∥}\;dP\left(\omega |∥\mu_{\omega }∥=n\right)\right)\cdot P\left(∥\mu_{\omega }∥=n|∥\mu_{\omega }∥>0\right)\\ \; & =\sum_{n=1}^{\infty }\left(\int_{\Omega }\frac{\mu_{\omega }\left(B\right)}{n}\;dP\left(\omega |∥\mu_{\omega }∥=n\right)\right)\cdot P\left(∥\mu_{\omega }∥=n|∥\mu_{\omega }∥>0\right)\\ \; & =\sum_{n=1}^{\infty }\frac{\int_{\Omega }\mu_{\omega }\left(B\right)\;dP\left(\omega |∥\mu_{\omega }∥)=n\right)}{n}\cdot P\left(∥\mu_{\omega }∥=n|∥\mu_{\omega }∥>0\right). \end{array} \end{array}$$

Now, the random variable $\mu_{\omega }\left(B\right)$ is Poisson distributed with mean $\mu_{\omega }\left(B\right)$ and the random variable $\mu_{\omega }\left(∁B\right)$ is Poisson distributed with mean $\mu_{\omega }\left(∁B\right)$ and these two random variables are independent. Furthermore, $\mu_{\omega }\left(B\right)+\mu_{\omega }\left(∁B\right)=\mu_{\omega }\left(A\right)=∥\mu_{\omega }∥$. When we condition on $∥\mu_{\omega }∥=n$ the distribution of $\mu_{\omega }\left(B\right)$ is binomial with mean $n\cdot \frac{\mu \left(B\right)}{∥\mu ∥}$. Hence,

(65) $$\begin{array}{c} \begin{array}{cc} \int_{\Omega }\frac{\mu_{\omega }\left(B\right)}{∥\mu_{\omega }∥}\;dP\left(\omega |∥\mu_{\omega }∥>0\right) & =\sum_{n=1}^{\infty }\frac{n\cdot \frac{\mu \left(B\right)}{∥\mu ∥}}{n}\cdot P\left(∥\mu_{\omega }∥=n|∥\mu_{\omega }∥>0\right)\\ \; & =\sum_{n=1}^{\infty }\frac{\mu \left(B\right)}{∥\mu ∥}\cdot P\left(∥\mu_{\omega }∥=n|∥\mu_{\omega }∥>0\right)\\ \; & =\frac{\mu \left(B\right)}{∥\mu ∥}. \end{array} \end{array}$$

 □

The theorem states that $\frac{\mu \left(B\right)}{∥\mu ∥}$ is the probability of observing a point in *B* if one first observes a multiset of points as an instance of a point process and then randomly selects one of the points from the multiset. This is not very different from random matrix theory, where one first calculates all eigenvalues of a random matrix and then randomly selects one of the eigenvalues. Wigner’s semicircular law states that such a randomly selected eigenvalue from a large random matrix has a distribution that approximately follows a semicircular law.

Proposition 2 holds for all point processes, but in Theorem 10 it is required that the point process is a Poisson point process. The following example shows there are point processes where Equation (63) does not hold.

**Example** **5.** *Let $\Omega =\{\omega_{1},\omega_{2}\}$ and assume that Pω1=Pω2=12. Let A={a,b}, and let $\omega \to \mu_{\omega }$ denote a process where $\mu_{\omega_{1}}=1\cdot \delta_{b}$ and $\mu_{\omega_{2}}=2\cdot \delta_{a}+1\cdot \delta_{b}$. The expectation measure of this process is $\mu =1\cdot \delta_{a}+1\cdot \delta_{b}$. Let B={b}. Then, the left-hand side of Equation (63) evaluates to*

(66) $$\frac{\mu \left(B\right)}{∥\mu ∥}=\frac{1}{2}.$$

*The right-hand side of Equation (63) evaluates to*

(67) $$\int_{\Omega }\frac{\mu_{\omega }\left(B\right)}{∥\mu_{\omega }∥}\;dP\left(\omega |∥\mu_{\omega }∥>0\right)=1\cdot \frac{1}{2}+\frac{1}{3}\cdot \frac{1}{2}=\frac{2}{3}.$$

The Poisson interpretation of normalized expectation measures carries over to conditional measures.

**Corollary** **1.** *Let P denote a probability measure on *Ω* and assume that $\omega \to \mu_{\omega }$ is a Poisson point process with expectation measure μ. Let A and B be subsets of A with $\mu \left(A\right)>0$. Then,*

(68) $$\mu \left(B|A\right)=\int_{\Omega }\mu_{\omega }\left(B|A\right)\;dP\left(\omega |\mu_{\omega }\left(A\right)>0\right).$$

**Proof.**  A conditional measure is the normalization of an expectation measure restricted to a subset.

(69) $$\begin{array}{cc} \mu \left(B|A\right) & =\frac{\mu \left(B\cap A\right)}{\mu \left(A\right)}=\frac{\mu_{\cap A}\left(B\right)}{∥\mu_{\cap A}∥}. \end{array}$$

The corollary is proved by applying Theorem 10 to the measure $\mu_{\cap A}$. The condition $\mu \left(A\right)>0$ will ensure that $P\left(\mu_{\omega }\left(A\right)>0\right)>0$.  □

Let μ be an expectation measure on $A_{1}$ and let *g* be a map from $A_{1}$ to $A_{2}$. Then, the induced measure $g\left(\mu \right)$ is also an expectation measure. If $g\left(\mu \right)\left(a_{2}\right)>0$, then a Markov kernel $\mu \left(\cdot |\cdot \right)$ from $A_{2}$ to $A_{1}$ is given by

(70) $$\mu \left(a_{1}|g\left(a\right)=a_{2}\right)=\frac{\mu_{\cap g^{-1}\left(a_{2}\right)}\left(a_{1}\right)}{g\left(\mu \right)\left(a_{2}\right)}.$$

With this Markov kernel, the measure μ can be factored as

(71) $$\mu \left(a_{1}\right)=\mu \left(a_{1}|g\left(a\right)=a_{2}\right)\cdot g\left(\mu \right)\left(g\left(a_{2}\right)\right).$$

### 4.5. Independence

The notion of independence plays an important role in the theory of randomness, so we need to define this notion in the context of expectation measures.

**Definition** **3.** *Assume that μ is a measure on A. For i=1,2 let $g_{i}$ denote the mappings $A\to A_{i}$. Then, we say that $g_{1}$ is independent of $g_{2}$ if $g_{1}\left(\mu_{\cap g_{2}\left(a\right)=a_{2}}\right)$ does not depend on $a_{2}\in A_{2}$.*

**Theorem** **11.** *Let μ be a measure on $A=A_{1}\times A_{2}$ with projections $g_{1}$ and $g_{2}$. Then, $g_{1}$ is independent of $g_{2}$ if and only if*

(72) $$\mu \left(\omega_{1},\omega_{2}\right)=\frac{\mu_{1}\left(\omega_{1}\right)\cdot \mu_{2}\left(\omega_{2}\right)}{∥\mu ∥}$$

*where $\mu_{1}$ and $\mu_{2}$ are the marginal measures on $A_{1}$ and $A_{2}$, respectively.*

**Proof.**  We have

(73) $$\begin{array}{c} \begin{array}{cc} \mu \left(\omega_{1},\omega_{2}\right) & =\mu \left(\omega_{1}|g\left(\omega \right)=\omega_{2}\right)\cdot \mu \left(g\left(\omega \right)=\omega_{2}\right)\\ & =\mu \left(\omega_{1}|g\left(\omega \right)=\omega_{2}\right)\cdot \mu_{2}\left(\omega_{2}\right). \end{array} \end{array}$$

Let $\tilde{\mu }$ denote the unital measure on $A_{1}$ given by $\tilde{\mu }\left(\omega_{1}\right)=\mu \left(\omega_{1}|g\left(\omega \right)=\omega_{2}\right)$. Then,

(74)μω1,ω2=μ˜ω1·μ2ω2,μ1ω1=∑ω2∈A2μω1,ω2(75)=∑ω2∈A2μ˜ω1·μ2ω2=μ˜ω1·μ2A2=μ˜ω1·μA,(76)μ1ω1μA=μ˜ω1.

 □

Note that Equation (72) is the standard way of calculating expected counts in a contingency table under the assumption of independence. Note also that Equation (72) can be rewritten as

(77) $$\frac{\mu \left(\omega_{1},\omega_{2}\right)}{∥\mu ∥}=\frac{\mu_{1}\left(\omega_{1}\right)}{∥\mu_{1}∥}\cdot \frac{\mu_{2}\left(\omega_{2}\right)}{∥\mu_{2}∥},$$

which is the well-known equation that states that for independent variables the joint probability is the product of the marginal probabilities.

### 4.6. Information Divergence for Expectation Measures

Let *P* and *Q* be discrete probability measures. Then, *Kullback–Leibler divergence* is defined by

(78) $$D\left(P∥Q\right)=\left\{\begin{array}{cc} \sum_{i}P\left(i\right)\ln\left(\frac{P\left(i\right)}{Q\left(i\right)}\right), & \;\mathrm{if}\;P⪯Q;\\ \infty , & \;\mathrm{else}. \end{array}\right.$$

For arbitrary discrete measures μ and ν, we define *information divergence* by extending Equation (78) via the following formula:

(79) $$D\left(\mu ∥\nu \right)=\sum_{i}\mu \left(i\right)\ln\left(\frac{\mu \left(i\right)}{\nu \left(i\right)}\right)-\mu \left(i\right)+\nu \left(i\right).$$

With this definition, information divergence becomes a Csiszár *f*-divergence, and it gives a continuous function from the cones of discrete measures to the lower reals $\vec{\left[0,\infty \right]}$.

**Proposition** **3** ([37] Thm. 5)**.** *Added in proof: This result was published after the submission of this paper. The submitted paper contained a less general result. Let μ and ν denote two σ-finite measures. Then,*

(80) $$D\left(Po\left(\mu \right)∥Po\left(\nu \right)\right)=D\left(\mu ∥\nu \right).$$

Note that, on the left-hand side is a KL-divergence for probability measures, while the right-hand side is an information divergence for expectation measures.

Information divergence is a Csiszár *f*-divergence, but it is also a Bregman divergence defined on the cone of discrete measures, and except for a constant factor it is the only Bregman divergence that is also a Csiszár *f*-divergence. On an alphabet with at least three letters, KL-divergence may (except for a constant factor) also be characterized as the only Bregman divergence that satisfies a data processing inequality for Markov kernels of unital measures, and there are a number of equivalent characterizations [38], if the alphabet has at least three letters. Here, we focus on the convex cone of measures rather than the simplex of probability measures. Therefore, it is interesting to note that information divergence has a characterization on a one-letter alphabet as a Bregman divergence that satisfies the following property called 1-homogenuity.

(81) $$D\left(\alpha \cdot \mu ∥\alpha \cdot \nu \right)=\alpha \cdot D\left(\mu ∥\nu \right).$$

**Theorem** **12.** *Assume that $d:R_{+}^{2}\to R_{0,+}$ is a function that satisfies the following conditions:*

- *$d\left(x,y\right)\geq 0$ with equality when x=y.*
- *$\sum_{i=1}^{n}d\left(x_{i},y\right)$ is minimal when $y=\bar{x}.$*
- *$d\left(\alpha \cdot x,\alpha \cdot y\right)=\alpha \cdot d\left(x,y\right)$ for all α>0.*

*Then, there exists a positive constant c such that $d\left(x,y\right)=c\cdot \left(x\ln\left(\frac{x}{y}\right)-\left(x-y\right)\right).$*

**Proof.**  The first two conditions imply that *d* is a Bregman divergence ([38], Proposition 4) so there exists a strictly convex function *g* such that $d\left(x,y\right)=g\left(x\right)-\left(g\left(y\right)+\left(x-y\right)\cdot g^{\prime }\left(y\right)\right).$ According to Property 3, we have

(82)dα·x,α·y=α·dx,y,(83)gα·x−gα·y+α·x−α·y·g′α·y=α·gx−gy+x−y·g′y.

We differentiate with respect to *y* and obtain

(84)−α·g′α·y−α·g′α·y−α2·y·g″α·y=α·−g′y+g′y+y·g″y,(85)α2·y·g″α·y=α·y·g″y,(86)α·y·g″α·y=y·g″y.

This holds for all α,y>0 so $g^{\prime \prime }\left(y\right)\cdot y=c$ for some constant c>0.

(87)g″y·y=c,(88)g″y=cy,(89)g′y=c·lny,(90)gy=c·ylny−y+k,

for some constant k. Hence,

(91) $$\begin{array}{c} \begin{array}{cc} d\left(x,y\right) & =c\left(x\ln\left(x\right)-x+k\right)-\left(c\left(y\ln\left(y\right)-y+k\right)+\left(x-y\right)\cdot c\cdot \ln\left(y\right)\right)\\ \; & =c\cdot \left(x\ln\left(x\right)-x+k-\left(y\ln\left(y\right)-y+k+\left(x-y\right)\cdot \ln\left(y\right)\right)\right)\\ \; & =c\cdot \left(x\ln\left(x\right)-x-y\ln\left(y\right)+y-\left(x-y\right)\cdot \ln\left(y\right)\right)\\ \; & =c\cdot \left(x\ln\left(\frac{x}{y}\right)-x+y\right). \end{array} \end{array}$$

 □

The following theorem can be proved in the same way as similar theorems in [33].

**Theorem** **13.** *Let P be a Bernoulli sum on $M\left(A\right)$ with expectation measure $\pi \left(P\right)=\mu$. Then,*

(92) DT1nP∗n∥Poμ→0

*for n→∞.*

Let C denote a convex set of measures. Then, $D\left(C∥\nu \right)$ is defined as $\inf_{\mu \in C}D\left(\mu ∥\nu \right)$. If $D\left(C∥\nu \right)<\infty$ and the measure $\mu^{*}\in C$ satisfies $D\left(\mu^{*}∥\nu \right)=D\left(C∥\nu \right)$, then $\mu^{*}$ is called the *information projection* of ν on C [39,40,41].

**Proposition** **4.** *Let ν be a measure of a finite alphabet A, and let C be a convex set of measures on A. If $\mu^{*}$ is the information projection of ν on C, then $Po\left(\mu^{*}\right)$ is the information projection of $Po\left(\nu \right)$ on the convex hull of the probability measures of the form $Po\left(\mu \right)$ where μ∈C.*

**Proof.**  The measure $\mu^{*}$ is the information projection of ν if and only if the following Pythagorean inequality

(93) $$D\left(\mu ∥\nu \right)\geq D\left(\mu ∥\mu^{*}\right)+D\left(\mu^{*}∥\nu \right)$$

is satisfied for all μ∈C ([41], Theorem 8). Now, we have

(94) $$\begin{array}{c} \begin{array}{cc} D\left(Po\left(\mu \right)∥Po\left(\nu \right)\right) & =D\left(\mu ∥\nu \right)\\ \; & \geq D\left(\mu ∥\mu^{*}\right)+D\left(\mu^{*}∥\nu \right)\\ \; & =D\left(Po\left(\mu \right)∥Po\left(\mu^{*}\right)\right)+D\left(Po\left(\mu^{*}\right)∥Po\left(\nu \right)\right). \end{array} \end{array}$$

Since a Pythagorean inequality is satisfied for $Po\left(\mu^{*}\right)$, it must be the information projection of $Po\left(\nu \right)$ on the convex hull of the distributions $Po\left(\mu \right)$ where μ∈C.  □

*The reversed information projection* is defined similar to the definition of the information projection [2,42,43,44]. If C is a convex set, then $D\left(\mu ∥C\right)$ is defined as $\inf_{\nu \in C}D\left(\mu ∥\nu \right)$. If $D\left(\mu ∥C\right)<\infty$, then $\hat{\nu }\in C$ is said to be the reversed information projection of μ on C if $D\left(\mu ∥\hat{\nu }\right)=D\left(\mu ∥C\right)$.

**Proposition** **5.** *Let μ be a measure of a finite alphabet A, and let C be a convex set of measures on A. Assume that $\hat{\nu }\in C$ and that $D\left(\mu ∥C\right)<\infty .$ Then, the probability measure $Po(\hat{\nu })$ is the reverse information projection of $Po\left(\mu \right)$ on the convex hull of the set of probability measures {Po(ν)∣ν∈C}.*

**Proof.**  According to the so-called four point property by Csiszár and Tusnády [42], the measure $\hat{\nu }$ is the reverse information projection of μ on C if and only if

(95) $$\sum_{\omega \in \Omega }\left(\frac{\mu \left(\omega \right)}{\hat{\nu }\left(\omega \right)}\cdot \nu \left(\omega \right)-\mu \left(\omega \right)+\hat{\nu }\left(\omega \right)-\nu \left(\omega \right)\right)\leq 0.$$

for all ν in C. The probability measure Po(μ) has outcome space $N_{0}^{\Omega }$. For $\vec{j}\in N_{0}^{\Omega }$ we will write $Po\left(\mu ,\vec{j}\right)$ as short for $\prod_{\omega \in \Omega }Po\left(P\left(\omega \right),j_{\omega }\right)$. We calculate

(96) $$\begin{array}{c} \begin{array}{cc} \sum_{\vec{j}\in N_{0}^{\Omega }}\frac{Po\left(\mu ,\vec{j}\right)}{Po\left(\hat{\nu },\vec{j}\right)}\cdot Po\left(\nu ,\vec{j}\right) & =\sum_{\vec{j}\in N^{\Omega }}\prod_{\omega \in \Omega }\frac{Po\left(\mu \left(\omega \right),j_{\omega }\right)}{Po\left(\hat{\nu }\left(\omega \right),j_{\omega }\right)}\cdot Po\left(\nu \left(\omega \right),j_{\omega }\right)\\ \; & =\prod_{\omega \in \Omega }\sum_{j\in N_{0}}\frac{Po\left(\mu \left(\omega \right),j\right)}{Po\left(\hat{\nu }\left(\omega \right),j\right)}\cdot Po\left(\nu \left(\omega \right),j\right)\\ \; & =\prod_{\omega \in \Omega }\sum_{j\in N_{0}}\frac{\frac{\mu {\left(\omega \right)}^{j}}{j!}\exp\left(-\mu \left(\omega \right)\right)}{\frac{\hat{\nu }{\left(\omega \right)}^{j}}{j!}\exp\left(-\hat{\nu }\left(\omega \right)\right)}\cdot \frac{\nu {\left(\omega \right)}^{j}}{j!}\exp\left(-\nu \left(\omega \right)\right)\\ \; & =\prod_{\omega \in \Omega }\sum_{j\in N_{0}}\frac{{\left(\frac{\nu \left(\omega \right)}{\hat{\nu }\left(\omega \right)}\cdot \nu \left(\omega \right)\right)}^{j}}{j!}\exp\left(-\mu \left(\omega \right)+\hat{\nu }\left(\omega \right)-\nu \left(\omega \right)\right)\\ \; & =\prod_{\omega \in \Omega }\exp\left(\frac{\mu \left(\omega \right)}{\hat{\nu }\left(\omega \right)}\cdot \nu \left(\omega \right)\right)\exp\left(-\mu \left(\omega \right)+\hat{\nu }\left(\omega \right)-\nu \left(\omega \right)\right)\\ \; & =\exp\left(\left(\sum_{\omega \in \Omega }\frac{\mu \left(\omega \right)}{\hat{\nu }\left(\omega \right)}\cdot \nu \left(\omega \right)-\mu \left(\omega \right)+\hat{\nu }\left(\omega \right)-\nu \left(\omega \right)\right)\right)\\ \; & \leq \exp\left(0\right)=1. \end{array} \end{array}$$

Therefore,

(97) $$\begin{array}{c} \begin{array}{cc} \sum_{\vec{j}\in N_{0}^{\Omega }}\left(\frac{Po\left(\mu ,\vec{j}\right)}{Po\left(\hat{\nu },\vec{j}\right)}\cdot Po\left(\nu ,\vec{j}\right)-Po\left(\mu ,\vec{j}\right)+Po\left(\hat{\nu },\vec{j}\right)-Po\left(\nu ,\vec{j}\right)\right) & \leq 1-1+1-1\\ \; & =0 \end{array} \end{array}$$

for all ν∈C.  □

## 5. Applications

In this section, we will present some examples of how expectation measures can be used to give a new way to handle some problems from statistics and probability theory.

### 5.1. Goodness-of-Fit Tests

Goodness-of-fit tests are often based on some Gaussian approximation. For small sample sizes and discrete distributions, such Gaussian approximations are sometimes problematic. Here, we shall see how our theory will help in one of the simplest possible setups. We will test whether a coin is fair, and we will perform an experiment where we count the number of heads and tails after tossing the coin a number of times. Let *X* denote the number of heads and let *Y* denote the number of tails. Our null hypothesis is that there is symmetry between heads and tails. Here, we will analyze the case when we have observed X=ℓ and Y=m.

Typically, one will fix the number of tosses so that X+Y=n, and assume that *X* has a binomial distribution with success probability p. First, we will look at an example wherein the null hypothesis states that p=12 and n=20.

The maximum likelihood estimate of *p* is ℓn. The divergence is

(98) Dbinn,ℓnbinn,12=n·ℓnlnℓn12+mnlnmn12.

We introduce the signed log-likelihood as

(99) Gnx=−2·Dbinn,xnbinn,1212,ifx<n2;+2·Dbinn,xnbinn,1212,ifx≥n2.

so that

(100) Dbinn,ℓnbinn,12=12Gnx2

**Figure 2:**
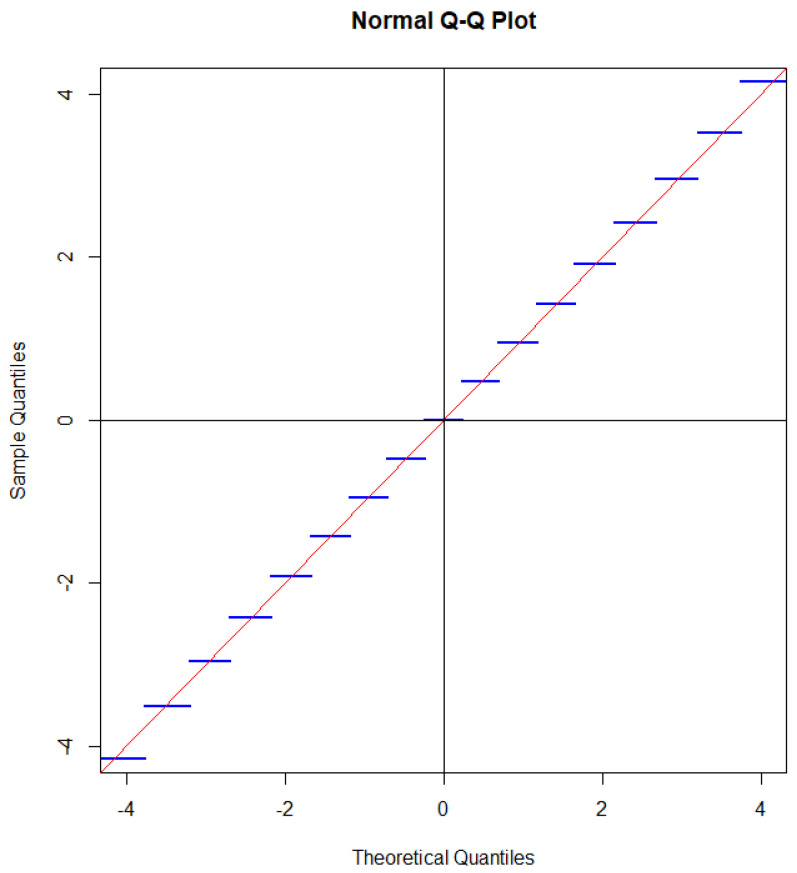
QQ-plot of a standard Gaussian distribution (red) against the distribution of $G_{20}\left(X\right),$ where *X* has a binomial distribution (blue) with n=20 and p=12.

In ([45], Cor 7.2), it is proved that

(101) $$\mathrm{Pr}\left(X<k\right)\leq \Phi \left(G_{n}\left(k\right)\right)\leq \mathrm{Pr}\left(X\leq k\right)$$

where Φ denotes the distribution function of a standard Gaussian distribution. A QQ plot with a Gaussian distribution on the first axis and the distribution of $G_{n}\left(X\right)$ on the second axis one obtains a staircase function with horizontal steps each intersecting the line x=y corresponding to a perfect match between the distribution of $G_{n}\left(X\right)$ and a standard Gaussian distribution, as illustrated in Figure 2.

Instead of using the Gaussian approximation, one could calculate tail probabilities exactly (Fisher’s exact test), but as we shall see below, one can even do better.

In expectation theory, it is more natural to assume that *X* and *Y* are independent Poisson distributed random variables with mean values λ and μ, respectively. In our analysis, the null hypothesis states that λ=μ.

Since $X+Y∼Po\left(\lambda +\mu \right)$, the maximum likelihood estimate of λ+μ is ℓ+m. Hence, the estimate of λ and μ are $\left(\ell +m\right)/2.$ Here, we define the random variable N=X+Y and n=ℓ+m. We calculate the divergence

(102) DPoℓ⊗Pom∥Pon2⊗Pon2=ℓlnℓn2+mlnmn2.

i.e., the same expression as in the classical analysis. Since *X* is binomial given that X+Y=n we have

(103) $$\mathrm{Pr}\left(X<k|N=n\right)\leq \Phi \left(G_{n}\left(k\right)\right)\leq \mathrm{Pr}\left(X\leq k|N=n\right),$$

**Figure 3:**
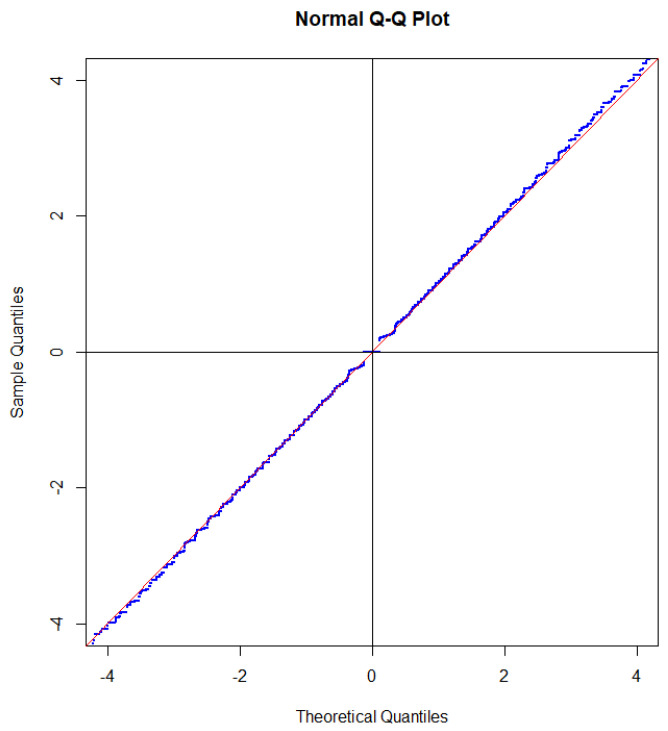
QQ-plot of a standard Gaussian distribution (red) against the distribution of $G_{N}\left(X\right)$ (blue), where *X* has a Poisson distributed with mean λ=10 and N=X+Y where *Y* is also Poisson distributed with mean 10 and *Y* is independent of *X*.

Since the distribution of $G_{N}\left(X\right)$ is close to a standard Gaussian distribution under the condition N=n, the same is true for $G_{N}\left(X\right)$ when we take the mean value over the Poisson distributed variable N. Since each of the steps intersects the straight line near the mid-point of the step, the effect of taking the mean value with respect to *N* is that the steps to a large extent cancel out, as illustrated in Figure 3.

The only step that partly survives the randomization is the step $G\left(X\right)=0$. For the binomial distribution, the length of this step is determined by PX=n2=0.1762. If the sample size is Poisson distributed, then the length of the step is determined as

(104)EPX=N2=∑neven20nn!exp−20·n!n2!22−n(105)=0.0898,

which is about half of the value for the binomial distribution. The reason for this is that the probability that a Poisson distributed random variable is even is approximately 12. If we tested p=13, we would obtain a step of length about one-third of the corresponding step for the binomial distribution. In a sense, testing p=12 gives the most significant deviation from a Gaussian distribution.

**Figure 4:**
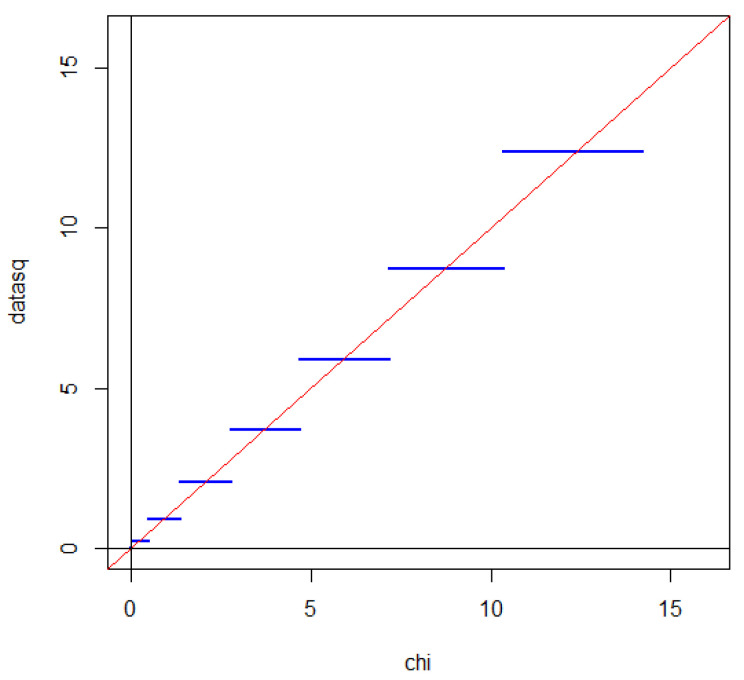
QQ-plot of the $\chi^{2}$-distribution with df=1 (red) against the distribution of the $G^{2}$-statistic for testing p=12 in $bin\left(20,p\right)$ (blue).

If we square $G\left(X\right)$, we obtain two times divergence, which is often called the $G^{2}$-statistic. Due to symmetry between head and tail, the intersection property is also satisfied when the distribution of the $G^{2}$-statistic is compared with a $\chi^{2}$-distribution [46]. This is illustrated in Figure 4.

**Figure 5:**
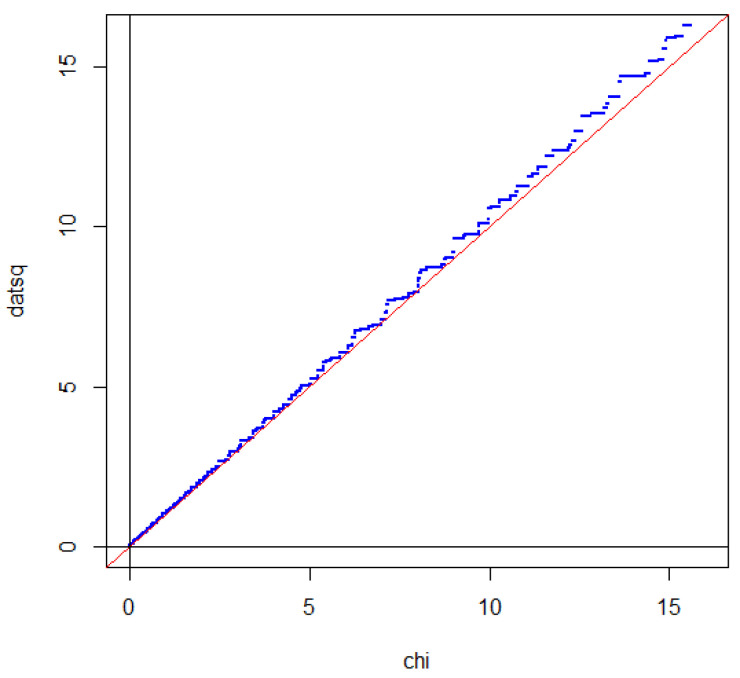
QQ-plot of the $\chi^{2}$-distribution with df=1 (red) against the distribution of the $G^{2}$-statistic (blue) for testing λ=μ based on $\hat{\lambda }+\hat{\mu }=20.$ We see that there is a tiny but systematic deviation from the straight line in that the values of $G^{2}$ are a little larger than predicted by the $\chi^{2}$-distribution.

For statistical analysis, one should not fix the sample size before sampling. A better procedure is to sample for a specific time so that the sample size becomes a random variable (Figure 5). In practice, this is often how sampling takes place and if the sample size is really random, it may even be misleading to analyze data as if *n* was fixed.

### 5.2. Improper Prior Distributions

Prior distributions play a major role in Bayesian statistics. A detailed discussion about how prior distributions can be determined in various cases is beyond the topic of this article. We will refer to [47] for a review of the subject including a long list of references. See [48] for a more information theoretic treatment of prior distributions. Here we shall just look at how the results of Section 4.4 will allow us to give an exact interpretation of conditional probabilities with respect to an *improper prior distribution*.

In Bayesian statistics, a justification of how posterior distributions are calculated is normally based on a probabilistic interpretation of the prior distribution. It is well-known that proportional prior distributions will lead to the same posterior distribution. For this reason, the total weight of the prior distribution is not important as long as the total weight is finite. If the total weight is finite, then the total weight can always be normalized in order to obtain a unital measure, and unital measures allow for a probabilistic interpretation within Bayesian statistics. A significant problem in Bayesian statistics is the use of improper prior distributions, i.e., prior distributions described by measures with infinite total mass. Such a prior is problematic in that it does not allow for a literal interpretation in Bayesian statistics. If we replace probability measures by expectation measures, this problem disappears. We will just give a simple example of how improper prior distributions can be given probabilistic interpretation using the Poisson interpretation of expectation measures.

Consider a statistical model where a random variable *X* has distribution given by the Markov kernel

(106) PX=μ−1=PX=μ+1=12.

**Figure 6:**
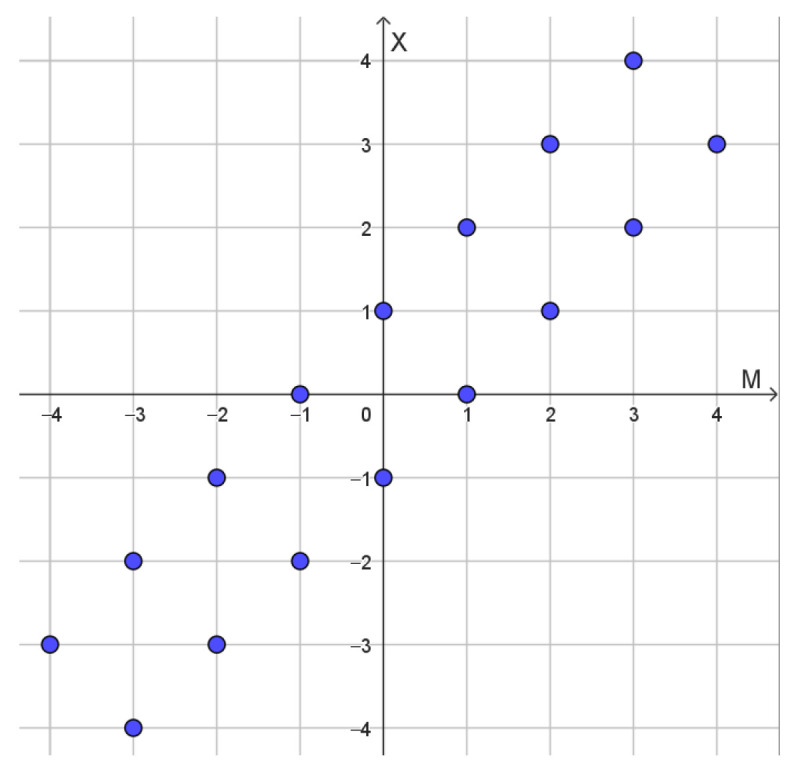
Support of the joint measure.

We assume that the mean value parameter μ is integer valued. We choose the counting measure times λ on Z as an improper prior distribution for the mean value parameter. There are a number of ways or arguing in favor of this prior distribution. For instance, the counting measure is invariant under integer translations. Except for a multiplicative constant translation, invariance uniquely determines the prior measure as a Haar measure. Combining λ times the counting measure on Z with the Markov kernel (106), we obtain a joint measure on $Z^{2}$ supported on the points illustrated in Figure 6.

Each point supporting the joint measure will have weight λ2. The marginal measure on *X* is λ times the counting measure on Z, as discussed in Section 4.4. Now, the joint measure can be factored into λ times the counting measure on Z and a Markov kernel

(107) PM=x−1=PM=x+1=12.

The Markov kernel (107) can be considered as the posterior distribution of *M* given that X=x.

These calculations can be justified within a probabilistic setting via point processes. First, the prior is represented by a Poisson point process with λ times the counting measure on Z as expectation measure. If the Markov kernel (106) is applied to this process, then we obtain a Poisson point process on the points in $Z^{2}$ illustrated in Figure 6 with λ2 times the counting measure as expectation measure. For an instance of this joint point process let $N_{x}$ denote the number of points with *x* as second coordinate. We will condition on X=x so we will assume that $N_{x}=n>0.$ Among these *n* points that all have second coordinate equal to *x*, we choose a random point according to a uniform distribution, i.e., each point is selected with probability 1n. The selected point has coordinates $\left(M,x\right)$ where M=x±1. We want to calculate the distribution of *M* for a given value of x. According to Corollary 1 we obtain PM=x±1∣X=x=12 and this is our posterior distribution.

This derivation works for any value of λ>0. In particular, we will obtain the same result if we thin the process by a factor α>0, i.e., if we replace λ by α·λ. The derivation involves selecting one out of $N_{x}$ points under the condition that $N_{x}\geq 1.$ If $N_{x}∼Po\left(\alpha \cdot \lambda \right)$, we have

(108) $$\begin{array}{c} \begin{array}{cc} P\left(N=1|N\geq 1\right) & =\frac{\alpha \cdot \lambda \exp\left(-\alpha \cdot \lambda \right)}{1-\exp\left(-\alpha \cdot \lambda \right)}\\ \; & =\frac{\alpha \cdot \lambda }{\exp\left(\alpha \cdot \lambda \right)-1}\\ \; & \to 1 \end{array} \end{array}$$

for α→0. Therefore, with high probability there will only be one point that has second coordinate *x* if the point process has been thinned with a small value of α. Hence, the step, where we randomly select one of the points, becomes almost obsolete if we thin the process sufficiently.

If the Poisson point process is a process in time, then there will be a first point for which X=x and the probability of M=m will be the probability that this first point satisfies M=m. With a process in time, one can introduce a stopping time that stops the process when X=x has been observed for the first time. This often simplifies the interpretation, but precisely this kind of reasoning goes wrong in many presentations of the double-slit experiment (Example 3). In this experiment, the point process is not a process in time because no arrival times for the photons are recorded. If one had precise time records, the energy and frequency uncertainty would destroy the interference pattern.

### 5.3. Markov Chains

The idea of randomizing the sample size has various consequences, and sometimes, this leads to great simplifications. In statistics and ergodic theory, an average is usually taken with respect to a uniform distribution, but from the point of view of expectation theory it is often more natural to take the mean with respect to a Poisson distribution. Here, we will see how this simplifies certain aspects of the theory of Markov chains.

Let Φ denote some Markov operator that generates a Markov chain. The time average is usually defined as

(109) $$\Phi_{n}=\frac{1}{n}\sum_{i=0}^{n-1}\Phi^{i}$$

Many properties of such time averages are known, but what complicates the matter is that the composition of $\Phi_{m}$ and $\Phi_{n}$ is not a time average. If we instead define

(110) $$\Psi_{t}=\sum_{i=0}^{\infty }\frac{t^{i}}{i!}\exp\left(-t\right)\cdot \Phi^{i}$$

then $\Psi_{t}$ is a Markov operator and $\Psi_{s}\circ \Psi_{t}=\Psi_{s+t}$. The Markov operators $\Psi_{t}$ generate a Markov process in continuous time, and this Markov process tend to have better properties than the original Markov chain. For instance, all recurrent states under Φ obtain positive transition probabilities under Ψ. The same idea was also used in [49] to prove that, if Φ is an affine map of a convex body into itself, then $\Psi_{t}$ converges to a retraction of the convex body to the set of fix points of the affine map Φ. Many theorems in probability involving averages should be revisited to see what the consequences are if the usual average is replaced by taking a weighted average with Poisson distributed weights.

### 5.4. Inequalities for Information Projections

Many algorithms used in probability theory and statistics are focused on unital measures in the sense that, at each time a non-unital measure is calculated, the next step will be to normalize the measure. Sometimes, there are good reasons for normalizing the measures, but there are also important cases wherein normalization introduces unnecessary complications. Here, we will illustrate this point with some results involving information projections. Such information projections appear as building blocks for some important algorithms in information theory and statistics like the Arimoto–Blahut algorithm and the EM algorithm.

Let $Q=\left(q_{1},q_{2},\ldots ,q_{n}\right)$ be a unital measure on a finite set and let *C* denote the convex set of unital measures such that the mean value of f(i) is ν. Then, the information projection *P* of *Q* on *C* can be determined by using Lagrange multipliers.

(111)lnP(i)Q(i)+P(i)P(i)−1=β·f(i)+γ·1,(112)P(i)Q(i)=expβ·f(i)+γ,(113)P(i)=expβ·f(i)·expγ·Q(i).

*The moment generating function* is defined by $Z\left(\beta \right)=\sum_{i}\exp\left(\beta \cdot f(i)\right)\cdot Q\left(i\right)$ and in order to obtain a unital measure we shall choose $\gamma =-\ln\left(Z(\beta )\right)$. Thus, *P* is the element in the *exponential family*

(114) $$P\left(i\right)=\frac{\exp\left(\beta \cdot f(i)\right)}{Z(\beta )}\cdot Q\left(i\right).$$

The mean value is $\frac{Z^{\prime }\left(\beta \right)}{Z(\beta )}$ and β should be chosen so that this equals ν.

If we drop the condition that the projection should be unital, we obtain simplified expressions. The Lagrange equation becomes

(115)lnPβ(i)Q(i)+Pβ(i)Pβ(i)−1=β·f(i),(116)Pβ(i)=expβ·f(i)Q(i),

and the mean value of this measure is $Z^{\prime }\left(\beta \right)$.

**Theorem** **14.** *Let Q be a unital measure on a finite set and let X be a random variable such that*

(117)∫XdQ=0,(118)∫X2dQ=1,(119)∫X3dQ<0.

*Then, there exists δ>0 such that*

(120) $$D\left(P∥Q\right)\geq \frac{1}{2}{\left(\int X\;dP\right)}^{2}$$

*for all measures satisfying $\int X\;dP\in \left[0,\delta \right].$*

**Proof.**  For a fixed value of ∫XdP the right-hand side is minimized for the distribution $P_{\beta }$ determined by (116), so it is sufficient to prove the inequality for $P=P_{\beta }$. Thus, we have to prove that

(121) $$\beta \cdot Z^{\prime \prime }\left(\beta \right)-Z\left(\beta \right)+\beta \geq \frac{1}{2}{\left(Z^{\prime }\left(\beta \right)\right)}^{2}$$

for 0≤β≤δ for some δ>0. We differentiate both sides, and it is sufficient to prove that

(122)β·Z″(β)≥Z′(β)·Z″(β),(123)β≥Z′(β),

because $Z^{\prime \prime }\left(\beta \right)>0$. We differentiate once more, and we have to prove that

(124) $$1\geq Z^{\prime \prime }\left(\beta \right).$$

Now, we differentiate one more time, and we have to prove that

(125) $$0\geq Z^{\prime \prime \prime }\left(\beta \right).$$

for 0≤β≤δ but this holds by continuity because $Z^{\prime \prime \prime }\left(0\right)=\int X^{3}\;dQ<0$.  □

Similar results hold for measures on infinite sets, but in these cases one should be careful to choose the parameters so that the information projection exists. In a number of important cases, the inequality above may be extended to all positive (or negative) values rather than positive (or negative) values in a neighborhood of zero. Some of these cases are mentioned below.

Hypergeometric distributions can be approximated by binomial distributions. A lower bound is given by the following inequality [50].

(126) $$D\left(P∥bin(n,p)\right)\geq \frac{{\left(\sum_{x=0}^{n}{\tilde{K}}_{2}\left(x;n,p\right)P\left(x\right)\right)}^{2}}{2}$$

where ${\tilde{K}}_{2}\left(x;n,p\right)$ is the second normalized Kravchuk polynomial. For hypergeometric distribution $hyp\left(N,K,n\right)$ with $\frac{K}{N}=p$, this lower bound can be written as

(127) $$D\left(hyp\left(N,K,n\right)∥bin\left(n,p\right)\right)\geq \frac{n(n-1)}{4{\left(N-1\right)}^{2}}$$

As demonstrated in [50], this lower bound is tight for fixed values of *n* and *p* and large values of the parameters *N* and *K*.

Binomial distributions can be approximated by Poisson distributions. For any distribution *P*, we have the following lower bound on information divergence.

(128) $$D\left(P∥Po\left(\lambda \right)\right)\geq \frac{{\left(\sum_{x=0}^{\infty }{\tilde{C}}_{2}^{\lambda }\left(x\right)P\left(x\right)\right)}^{2}}{2}$$

where ${\tilde{C}}_{2}^{\lambda }\left(x\right)$ denotes the second normalized Poisson–Charlier polynomial. If *P* is binomial $bin\left(n,p\right)$ and λ=np, then we obtain the inequality

(129) $$D\left(bin\left(n,p\right)∥Po\left(\lambda \right)\right)\geq \frac{p^{2}}{4}$$

and this inequality is tight for fixed λ and *n* tending to infinity [33,51,52].

In the central limit theorem the rate of convergence is primarily determined by the skewness and if the skewness is zero it is primarily determined by the excess kurtosis. If the skewness is non-zero, then lower bounds on divergence involve both skewness and kurtosis, so for simplicity we will only present the case where the skewness is zero and the excess kurtosis is negative. For all measures *P* for which the integral $\int {\tilde{H}}_{4}\left(x\right)\;dP\leq 0$ where ${\tilde{H}}_{4}$ denotes the fourth normalized Hermite polynomial, we have

(130) $$D\left(P∥N(o,1)\right)\geq \frac{{\left(\int {\tilde{H}}_{4}\left(x\right)\;dPx\right)}^{2}}{2}.$$

If *P* is a unital measure with variance excess kurtosis κ, then according to ([53], Theorem 7) this inequality reduces to

(131) $$D\left(P∥N(0,1)\right)\geq \frac{\kappa^{2}}{48}.$$

Lower bounds for the rate of convergence have been discussed in the paper [53] where this and similar inequalities were treated in great detail.

For all these inequalities, it gives simplifications if we do not require that the measures are unital. The hard part is to prove that the inequalities do not only hold in a neighborhood of zero, but that they hold for all values of interest. This hard part of the proofs is still hard without the assumption that the measures are unital, but it should be noted that the hard part is not relevant if we are only interested in lower bounds on the rate of convergence.

## 6. Discussion and Conclusions

Expectation theory may be considered an alternative to Kolmogorov’s theory of probability. Still, it may be better to view the two theories as two ways of describing the same situations using measure theory. The language of probability theory focuses on experiments where a single outcome is classified according to mutually exclusive classes. The language of expectation theory focuses on experiments where the results are given as tables of frequencies. There will be no inconsistency if the two languages are mixed. Expectation measures can be understood within probability theory, as is already the case in the theory of point processes. If our understanding of randomness is based on expectation measures, then an expectation measure can be interpreted as the expectation measure of a process in a larger outcome space.

In this paper, we have only worked out the basic framework and interpretation of expectation theory. This opens up a significant number of new research questions. For instance, it should be possible to justify the use of Haar measures as prior distributions rigorously in the same way as Haar probability measures on compact groups can be justified as being probability measures that maximize the rate-distortion function [54]. In expectation theory, it is natural to consider sampling with a randomized sample size. Since many results in probability theory are formulated for fixed sample sizes, there is a significant amount of work to be done to generalize the results to cases with random sample sizes. Our present results suggest that simpler or stronger results can be obtained, but in many cases new techniques may be needed.

In this paper, we discussed finite expectation measures and valuations on topological spaces and downsets of posets that satisfy the DCC. The valuation approach is more general, but it is an open research question which category is most useful for further development of expectation theory.

Quantum information theory can be based on generalized probabilistic theories. In these theories, a measurement is defined as something that maps a preparation into a probability measure. A *state* is defined as an equivalence classes of preparations that cannot be distinguished be any measurement. With a shift from probability measures to expectation measures, one should similarly change the focus in quantum theory from states that are represented by density operators (positive operators with trace 1) to positive trace class operators. Thus, the focus should shift from the convex state space to the state cone. For applications in quantum information theory, it is still too early to say what the impact will be, but shifting the focus away from mutually exclusive events may circumvent some of the paradoxes that have haunted the foundation of quantum theory for more than a century.

Below is a list of concepts from probability theory and standard quantum information theory and how they relate to the concepts that have been introduced in the present paper.
**Probability theory****Expectation theory**ProbabilityExpected valueOutcomeInstanceOutcome spaceMultiset monadP-valueE-ValueProbability measureExpectation measureBinomial distributionPoisson distributionDensityIntensityBernoulli random variableCount variableEmpirical distributionEmpirical measureKL-divergenceInformation divergenceUniform distributionPoisson point processState spaceState cone

## Data Availability

No new data were created or analyzed in this study. Data sharing is not applicable to this article.

## Abbreviations

The following abbreviations are used in this manuscript:

bin	Binomial distribution
DCC	Descending chain condition
E-statistic	Evidence statistic
E-value	Observed value of an E-statistic
hyp	Hypergeometric distribution
IID	Independent identically distributed
KL-divergence	Information divergence restricted to probability measures
MDL	Minimum description length
Mset	Multiset
N	Gaussian distribution
PM	Probability measure
Po	Poisson distribution
mset	Multiset
Poset	Partially ordered set
Pr	Probability
